# *Bacilli-*Mediated Degradation of Xenobiotic Compounds and Heavy Metals

**DOI:** 10.3389/fbioe.2020.570307

**Published:** 2020-10-09

**Authors:** Pankaj Kumar Arora

**Affiliations:** Department of Microbiology, Babasaheb Bhimrao Ambedkar University, Lucknow, India

**Keywords:** 4-Chloro-2-nitrophenol, naproxen, polycyclic aromatic hydrocarbons, cypermethrin, ibuprofen, *Bacillus*

## Abstract

Xenobiotic compounds are man-made compounds and widely used in dyes, drugs, pesticides, herbicides, insecticides, explosives, and other industrial chemicals. These compounds have been released into our soil and water due to anthropogenic activities and improper waste disposal practices and cause serious damage to aquatic and terrestrial ecosystems due to their toxic nature. The United States Environmental Protection Agency (USEPA) has listed several toxic substances as priority pollutants. Bacterial remediation is identified as an emerging technique to remove these substances from the environment. Many bacterial genera are actively involved in the degradation of toxic substances. Among the bacterial genera, the members of the genus *Bacillus* have a great potential to degrade or transform various toxic substances. Many *Bacilli* have been isolated and characterized by their ability to degrade or transform a wide range of compounds including both naturally occurring substances and xenobiotic compounds. This review describes the biodegradation potentials of *Bacilli* toward various toxic substances, including 4-chloro-2-nitrophenol, insecticides, pesticides, herbicides, explosives, drugs, polycyclic aromatic compounds, heavy metals, azo dyes, and aromatic acids. Besides, the advanced technologies used for bioremediation of environmental pollutants using *Bacilli* are also briefly described. This review will increase our understanding of *Bacilli*-mediated degradation of xenobiotic compounds and heavy metals.

## Introduction

The genus *Bacillus* belongs to the family *Bacillaceae* that comprises of 293 species/subspecies (Patel and Gupta, 2020). This genus is characterized by a group of rod-shaped, Gram-positive, aerobic, or facultatively anaerobic, endospore-forming bacteria (Patel and Gupta, 2020). Members of the genus *Bacillus* are ubiquitous; they have been isolated from a variety of sources including soil, sewage sludge (Demharter and Hensel, 1989), ocean sediments (Ruger et al., 2000), saline water (Smibert and Krieg, 1994). They have the exceptional ability to grow very rapidly in high densities as well as to tolerate adverse environmental conditions. The genus *Bacillus* includes both non-pathogenic (free-living) and pathogenic (parasitic) species. Few examples of non-pathogenic species are *Bacillus subtilis, B. licheniformis, B. amyloliquefaciens*, and *B. pumilus*, which are closely related to each other. The pathogenic strains include *B. anthracis*, which causes anthracis in human beings and *B. cereus* that causes food poisoning (Claus and Berkeley, 1986).

*Bacilli* constitute a versatile group of bacteria, which have many applications in the field of health, environment, and agriculture. They produce several secondary metabolites including antibiotics and biosurfactants (Caulier et al., 2019). Furthermore, they are potential sources of industrial enzymes including lipases, proteases, alpha-amylase, and the BamH1 restriction enzyme (Latorre et al., 2016). Few species of *Bacillus* including *B. thuringiensis*, and some strains of *B. sphaericus* have insecticidal properties (Palma et al., 2014). Using the genetic engineering approach, the genes encoding insecticidal proteins in *B. thuringiensis* have been incorporated into corn and cotton plants to generate insect-resistance genetically modified crops (Jouzani et al., 2017). Some *Bacillus* species are ideal candidates for biological control due to their antagonistic activities against fungal and some bacterial pathogens (Wulff et al., 2002).

*Bacilli* are considered as potential bioremediator agents, which are capable of degrading several toxic substances (Arora et al., 2016; Singh and Singh, 2016; Xiao et al., 2017). Earlier studies have also been reported degradation of various xenobiotic compounds and heavy metals by the members of genus *Bacillus* (Birolli et al., 2016; Upadhyay et al., 2017; Arora et al., 2018; Díez-Méndez et al., 2019). Wang et al. (2019) reported the efficient biodegradation of petroleum hydrocarbons by *B. subtilis* BL-27. Viesser et al. (2020) isolated new petroleum-degrading strains of *B. thuringiensis* and *B. subtilis* from the rhizosphere of *Panicum aquaticum*. Both of these strains were able to utilize petroleum hydrocarbons as their sole source of carbon and energy. Bonifer et al. (2019) reported that *B. pumilus* B12 degrades poly-lactic acid that is the second most common biodegradable polymer found in commercial plastics. The transformation of 4-chloro-2-nitrophenol was extensively studied in many *Bacilli* (Arora et al., 2018). The ability of *Bacilli* to degrade polycyclic aromatic compounds, drugs, dyes, explosives have also been reported in the literature (Singh and Singh, 2016; Górny et al., 2019). These data indicated that *Bacilli* play a significant role in the biodegradation of toxic substances.

So far, several reviews have been published dealing with the biotechnological application of *Bacilli* (Bunk et al., 2010; Kumar et al., 2013; Jouzani et al., 2017; Sansinenea, 2019). Kumar et al. (2013) reviewed the significance of *Bacilli* for the production of biofuels, polyhydroxyalkanoates, and bioactive molecules. Sansinenea and Ortiz (2011) described the importance of secondary metabolites produced by *Bacilli*. Bunk et al. (2010) summarized the industrial applications of *Bacillus megateriunm* and other *Bacilli*. Sansinenea (2019) discussed the plant growth-promoting activities of *Bacilli*. Even though *Bacill i*are highly involved in biodegradation of various natural and xenobiotic compounds, a review on the biodegradation potential of *Bacilli* is rare. In the last decade, several researchers have been investigated the degradation abilities of *Bacilli* toward many toxic compounds. This review aims to summarize the role of *Bacilli* in the biodegradation process of various xenobiotic compounds and heavy metals.

## Role of *Bacillus* Species in Biodegradation

Table 1 summarizes the role of various *Bacilli* in biodegradation of dyes, pesticides, herbicides, chlorophenols, nitrophenols, chloronitrophenols, heavy metals, drugs, explosives, crude oil waste, plastics, alkaline lignin, and other natural compounds. One of the following processes may involve in the degradation of toxic compounds by *Bacilli*: (i) Complete mineralization of toxic compounds, (ii) Co-metabolism of xenobiotics compounds. The mineralization involves complete utilization of toxic compounds by a *Bacillus* strain which utilized them as its sole source of carbon energy and converts them into CO_2_ and water (Arora et al., 2018). In the co-metabolism, *Bacilli* transform chemical compounds into other compounds which generally less toxic than parent compounds. Co-metabolism-based bioremediation is a non-growth linked biological process in which bacteria convert environmental pollutants to other substances in the presence of carbon source or growth substrate (Hazen, 2010). In this process, bacteria do not depend on the pollutants for growth and use non-specific enzymes to degrade environmental pollutants that do not support their growth (Hazen, 2010). In this section, the biodegradation potential of *Bacilli* toward a variety of xenobiotic compounds and heavy metals is discussed.

**Table 1:** A list of members of the genus *Bacillus*, which have biodegradation potential.

**S. No**.	**Bacteria strain**	**Compounds**	**Remarks**	**References**
**A. Decolorization and transformation of Dyes**				
1.	*Bacillus aryabhattai* LN01	Toluidine Blue	Decolorized Toluidine Blue in Lacaase-like and azoreductase-like reactions	Díez-Méndez et al., 2019
2	*Bacillus aryabhattai* LN08	Toluidine Blue, Remazol, and Brilliant Blue	Decolorized Toluidine Blue in Lacaase-like reaction and Remazol Brilliant Blue in Peroxidase-like reaction	Díez-Méndez et al., 2019
3.	*Bacillus aryabhatta*i LN09	Congo Red, Toluidine Blue, and Remazol Brilliant Blue	Decolorized Congo Red in Laccase-like reaction; Toluidine Blue in Azoreductase-like reaction and Remazol Brilliant Blue in Peroxidase like reaction	Díez-Méndez et al., 2019
4.	*Bacillus aryabhattai* LN10	Toluidine Blue	Decolorized Toluidine Blue in Laccase-like and Azoreductase-like reactions	Díez-Méndez et al., 2019
5.	*Bacillus aryabhattai* LN15	Congo Red, and Toluidine Blue	Decolorized Congo Red and Toluidine Blue in Laccase-like and Azoreductase-like reactions	Díez-Méndez et al., 2019
6.	*Bacillus aryabhattai* LN16	Congo Red	Decolorized Congo Red by Peroxidase-like reaction	Díez-Méndez et al., 2019
7.	*Bacillus megaterium* LN30	Congo Red	Decolorized Congo red by Azoreductase-like reaction	Díez-Méndez et al., 2019
8.	*Bacillus aryabhattai* LN37	Congo Red, Toluidine Blue, and Remazol Brilliant Blue	Decolorized Congo red in Laccase like reaction; Remazol Brilliant Blue in Azoreductase-like reaction and Toluidine Blue in Laccase-like and Azoreducatase like reactions	Díez-Méndez et al., 2019
9.	*Bacillus aryabhattai* LN39	Remazol Brilliant Blue, and Toluidine Blue	Decolorized Remazol Brilliant Blue and Toluidine Blue in Azoreductase-like reaction.	Díez-Méndez et al., 2019
10.	*Bacillus aryabhattai* LN41	Remazol Brilliant Blue and Toluidine Blue	Decolorized Remazol Brilliant Blue and Toluidine Blue in Azoreductase like reduction	Díez-Méndez et al., 2019
11.	*Bacillus aryabhattai* LN49	Remazol Brilliant	Decolorized Remazol Brilliant Blue in Laccase-like reaction	Díez-Méndez et al., 2019
12.	*Bacillus aryabhattai* LN61	Toluidine Blue	Decolorized Toluidine Blue in Azoreductase-like reaction	Díez-Méndez et al., 2019
13.	*Bacillus aryabhattai* LN84	Congo Red, and Remazol Brilliant Blue	Decolorized Congo Red in Laccase-like reaction and Remazol Brilliant Blue in Azoreductase and Laccase-like reactions	Díez-Méndez et al., 2019
14.	*Bacillus aryabhattai* LN87	Toluidine Blue	Decolorized Toluidine Blue in Azoreductase and Laccase-like reactions	Díez-Méndez et al., 2019
15.	*Bacillus aryabhattai* LN88	Toluidine Blue and Remazol Brilliant Blue	Decolorized Toluidine Blue in Peroxidase-like and Azoreducase-like reactions and Remazol Brilliant Blue in Azoreucatase-like reactions	Díez-Méndez et al., 2019
16.	*Bacillus aryabhattai* LN90	Congo Red, Toluidine Blue, and Remazol Brilliant Blue	Decolorized Congo red in Laccase-like reaction; Toluidine Blue in Laccase-like and Azoredutase-like reactions and Remazol Brilliant Blue in Azoreductase-like reaction.	Díez-Méndez et al., 2019
17.	*Bacillus* sp. VUS	Brown 3REL	Transformed into 6,8-dichloro-quinazoline-4-ol and cyclopentanone.	Dawkar et al., 2008
18.	*Bacillus* sp. OY1-2	Red B dye	Rapid biodegradation of Red B dye was observed in anoxic conditions as compared to aerobic conditions.	Li et al., 2004
19.	*Bacillus firmus*	Reactive Blue 160	Decolorized dye (500 mg/l) and detoxify it.	Barathi et al., 2020
20.	*Bacillus megaterium* KY848339.1	Acid red 337 dye	Degraded it via small aliphatic compounds and CO2	Ewida et al., 2019
21.	*Bacillus* sp. BDN2	Reactive Blue 160	Degraded 65% dye	Balapurea et al., 2014
22.	*Bacillus* sp. BDN7	Reactive Blue 160	Degraded 80% within 12 h	Balapurea et al., 2014
23.	*Bacillus* sp. BDN8	Reactive Blue 160	Degraded 75% within 18 h	Balapurea et al., 2014
24.	*Bacillus* megaterium NCIM 2054	Disperse Red 73 dye	61% dye decolorization within 48 h	Kadam et al., 2014
25.	*Bacillus cereus* HJ-1	Reactive Black B	Decolorized dye and detoxify it.	Liao et al., 2013
26.	*Bacillus* sp. YZU1	Reactive Black 5	95% dye decolorization was observed in 120 h	Wang et al., 2013
27.	*Bacillus* sp. AK1	Metanil Yellow	Degraded 200 mg/l dye within 27 h	Anjaneya et al., 2011
28.	*Bacillus* odyssey SUK3	Reactive blue 59	Decolorized dye (50 mg/l) completely within 60 h	Patil et al., 2008
29.	*Bacillus* cereus DC11q	Malachite green	Degraded to 4,4′-bis(dimethylamino)benzophenone and benzophenone	Deng et al., 2008
30.	*Bacillus cereus*DC11q	Acid Blue 25	95–98% dye (100 μM) decolorization within 6 h under anaerobic conditions	Deng et al., 2008
31.	*Bacillus cereus* DC11q	Basic Blue X-GRRL	Degraded via reduction of azo bonds.	Deng et al., 2008
32.	*Bacillus fusiformis* KMK5	Disperse Blue 79, and Acid Orange 10	Complete mineralization of dyes at the concentration of 1.5 g/L was observed within 48 h	Kolekar et al., 2008
33.	*Bacillus subtilis* IFO 13719	Crystal Violet	Decolorized via 4,4'-bis(dimethylamino)benzophenone	Yatome et al., 1991
34.	*Bacillus megaterium*	Azo dye	Decolorized 98% dye	Shah et al., 2013
35.	*Bacillus cereus*	Azo dye	Decolorized 95% dye	Shah et al., 2013
36.	*Bacillus pseudomycoides*	Acid Black 24	96% of dye decolorization was achieved within 24 h.	Kumar et al., 2019
37.	*Bacillus subtilis*	Disperse yellow 211	80% dye (100 mg/) decolorization observed under optimum conditions.	Sharma et al., 2009
38.	*Bacillus subtilis*	Crystal violet	Decolorized dye (100 mg/l) effectively at pH 8 and temperature 35° C	Kochher and Kumar, 2011
39.	*Bacillus cohnii* MTCC 3616	Direct Red-22	95% dye decolorization (5,000 mg l^−1^) was observed at 37° C and pH 9 in 4 h	Prasad and Rao, 2013
40.	*Bacillus firmus*	CI Direct Red 80	Decolorized 50 mg/L of dye under anoxic conditions within 12 h	Ogugbue et al., 2012
41.	*Bacillus licheniformis*	Reactive Red 2	Transformed it into 2, 4-dichloro-6-[(1H-indazol-5-ylimino)-methyl]-phenol, benzene sulfonamide, 1H indole and urea as final metabolites	Sudha and Balagurunathan, 2013
42.	*Bacillus megaterium*	Remazol Blue	Decolorized up to 5 mg/ml	Joshi et al., 2013
43.	*Bacillus subtilis*	RED M5B	Decolorization by the activity of peroxidase	Gunasekar et al., 2013
44.	*Bacillus* sp. VUS	Orange T4LL	transforms it into 4-methyl-2-o-tolylazo-benzene-1,3-diamine and [3-(phenyl-hydrazono)-cyclohexa-1,4-dienyl]-methanol	Dawkar et al., 2010
45.	*Bacillus flexus*	Remazol Black	Decolorized 100% of dye within 24 h	Saini et al., 2018
46.	*Bacillus flexus*	Direct Blue	Decolorized 100% of dye within 24 h	Saini et al., 2018
47.	*Bacillus flexus*	Acid Orange	Decolorized 100% of dye within 24 h	Saini et al., 2018
**B. Biodegradation of Pesticides, Herbicides, and Insecticides**				
48.	*Bacillus subtilis* strain 1D	Cypermethrin	Completely metabolized via cyclododecylamine, phenol, 3-(2,2-dichloroethenyl 2,2-dimethyl cyclopropane carboxylate,1-decanol, chloroacetic acid, acetic acid, cyclopentan palmitoleic acid, and decanoic acid	Gangola et al., 2018
49.	*Bacillus* sp. strain SG2	Cypermethrin	Metabolized via Phenoxybenzaldehyde,2,2,3,3 tetramethylcyclopropanecarboxylic acid 4-propylbenzoate, 4-propylbenzaldehyde, phenol M-tert-butyl, and 1-dodecanol,	Pankaj et al., 2016
50.	*Bacillus subtilis* BSF01	Cypermethrin	Metabolized via Phenoxybenzaldehyde, 2,2,3,3 tetramethylcyclopropanecarboxylic acid	Xiao et al., 2015
51.	*Bacillus* sp. AKD1	Cypermethrin	Transformed Cypermethrin in presence of heavy metals	Tiwary and Dubey, 2016
52.	*Bacillus* sp. ISTDS2	Cypermethrin	Metabolized Cypermethrin in soil microcosm via formation of cyclopropane, carboxylic acid, hydroxyacetonitrile, and benzene ethanamine	Sundaram et al., 2013
53.	*Bacillus licheniformis* B-1	Cypermethrin	Degraded via 3-phenoxybenzoic acid	Lai et al., 2012
54.	*Bacillus thuringiensis* ZS-19	Cyhalothrin	Degraded via α-hydroxy-3-phenoxy-benzeneacetonitrile, 3-phenoxyphenyl acetonitrile, N-(2-isoproxy-phenyl)-4-phenoxy-benzamide, 3-phenoxybenzaldehyde, 3-phenoxybenzoate, and phenol	Chen et al., 2015
55.	*Bacillus cereus* PU	Malathion	Degraded via malathion monocarboxylic and dicarboxylic acid	Singh et al., 2012
56.	*Bacillus thuringiensis* MOS-5	Malathion	Degraded via malathion monocarboxylic and dicarboxylic acid	Zeinat et al., 2008
57.	*Bacillus megaterium* MCM B-423	Monocrotophos	Degraded into carbon dioxide, ammonia, and phosphates	Bhadbhade et al., 2002
58.	*Bacillus* sp. N1	Metribuzin	Used as a nitrogen source.	Zhang et al., 2014
59.	*Bacillus alkalinitrilicus*	Imidacloprid	Degraded via 6-chloronictinic acid nitrosamine	Sharma et al., 2014
60.	*Bacillus subtilis* Y242	Chlorpyrifos	96% degraded within 48 h	El-Helow et al., 2013
61.	*Bacillus pumilus* NY97-I	Carbendazim	87.76% degradation	Zhang et al., 2009
62.	*Bacillus cereus* WD-2	Prochloraz-manganese	90.7% degradation at pH 8.	Jiang et al., 2019
63.	*Bacillus* sp. DG-02	Fenpropathrin	Transformed into 3,4-dihydroxybenzoic acid, 3,4-dimethoxyphenol, and phenol	Chen et al., 2014
64.	*Bacillus aryabhattai* strain VITNNDJ5	Monocrotophos	Degraded via three routes.	Dash and Osborne, 2020
65.	*Bacillus firmus*	Fipronil	Degraded via fipronil sulfide, fipronil sulfone and fipronil amide.	Mandal et al., 2013
66.	*Bacillus* sp. TAP-1	Triazophos	Co-metabolized via hydrolyzing insecticide triazophos	Tang and You, 2012
67.	*Bacillus pumilus* W1	Organophosphates	Hydrolysis of organophosphates by enzyme encoding by *opdA*	Ali et al., 2012
68.	*Bacillus subtilis* DR-39	Profenofos	4-Bromo-2-chlorophenol was identified as a metabolite	Salunkhe et al., 2013
69.	*Bacillus subtilis* CS-126,	Profenofos	4-Bromo-2-chlorophenol was identified as a metabolite	Salunkhe et al., 2013
70.	*Bacillus subtilis* TL-171	Profenofos	4-Bromo-2-chlorophenol was identified as a metabolite	Salunkhe et al., 2013
71.	*Bacillus subtilis* TS-204	Profenofos	4-Bromo-2-chlorophenol was identified as a metabolite	Salunkhe et al., 2013
72.	*Bacillus* sp. strain C5	Methyl Parathion	Hydrolyzedmethyl parathion to 4-nitrophenol and other metabolites	Hao et al., 2014
73.	*Bacillus pumilus* C2A1	Chlorpyrifos	Degraded via 3,5,6-trichloro-2-pyridinol	Anwar et al., 2009
74.	*Bacillus subtilis* MTCC 8561	Endosulfan and Endosulfan sulfate	Used as sulfur source and transformed to endosulfan diol and endosulfan lactone	Kumar et al., 2014
75.	*Bacillus subtilis* HB-6	Atrazine	Mineralized via hydroxyatrazine, cyanuric acid, and urea	Wang et al., 2014
76.	*Bacillus badius* ABP6	Atrazine	Optimum conditions of the atrazine degradation were determined	Khatoon and Rai, 2020
77.	*Bacillus megaterium* strain Q3	Quinclorac	Transformed to 3, 7-dichloro-8-methyl-quinoline, 3-chlorin-8-quinoline-carboxylic and 8-quinoline-carboxylic	Liu et al., 2014
78.	*Bacillus licheniformis* CY-012	Fenvalerate	Co-metabolized via α-isopropyl-4-chlorobenzene acetic acid, 4-chlorobenzene acetic acid, 3-phenoxybenzyl alcohol, phenol, and benzoic acid.	Tang et al., 2018
79.	*Bacillus* sp. 4T	Esfenvalerate	Transformed to (i) 3-2-(4-chlorophenyl)-3-methylbutyric acid), (ii) phenoxybenzoic acid, (iii) 2-(3-hydroxyphenyl)acetic acid	Birolli et al., 2016
80.	*Bacillus* sp. 2B	Esfenvalerate	Transformed to (i) 3-2-(4-chlorophenyl)-3-methylbutyric acid), (ii) phenoxybenzoic acid, hydroxy phenoxybenzoic acid and 3-hydroxybenzoic acid(iii) 2-(3-hydroxyphenyl)acetic acid	Birolli et al., 2016
81.	*Bacillus* sp. P5CBNB	Esfenvalerate	Transformed to (i) 3-2-(4-chlorophenyl)-3-methylbutyric acid), (ii) phenoxybenzoic acid, hydroxy phenoxybenzoic acid	Birolli et al., 2016
82.	*Bacillus* sp. CBMAI 1833	Esfenvalerate	Transformed to (i) 3-2-(4-chlorophenyl)-3-methylbutyric acid), (ii) phenoxybenzoic acid, hydroxy phenoxybenzoic acid (iii) 2-(3-hydroxyphenyl)acetic acid	Birolli et al., 2016
83.	*Bacillus* sp. DG-2	3-phenoxybenzoic acid	Degraded via 3-(2-methoxyphenoxy) benzoic acid, protocatechuate, phenol, and 3,4-dihydroxy phenol.	Chen et al., 2012a
**C. Biodegradation and Biotransformation of Chlorophenol, Nitrophenol, and Chloronitrophenol**				
84.	*Bacillus licheniformis* strain SL10	2,4-Dichlorophenol	Degradation occurred via meta cleavage pathway of catechol or chlorocatechol	Chris Felshia et al., 2020
85.	*Bacillus* sp. MW-1	4-chloro-2-nitrophenol	Transformed to 5-chloro-2-methyl benzoxazole	Arora and Jain, 2012
86.	*Bacillus* subtilis RKJ 700	4-chloro-2-nitrophenol	Transformed to 5-chloro-2-methyl benzoxazole	Arora, 2012
87.	*Bacillus* cereus PC-1	4-chloro-2-nitrophenol	Decolorized up to concentration of 1.0 mM	Arora et al., 2016
88.	*Bacillus toyonensis* PC-2	4-chloro-2-nitrophenol	Decolorized up to a concentration of 0.9 mM	Arora et al., 2016
89.	*Bacillus thuringiensis* PC-3	4-chloro-2-nitrophenol	Decolorized up to a concentration of 1.0 mM	Arora et al., 2016
90.	*Bacillus firmus* PC-4	4-chloro-2-nitrophenol	Decolorized up to a concentration of 0.8 mM	Arora et al., 2016
91.	*Bacillus koreensis* PC-5	4-chloro-2-nitrophenol	Decolorized up to concentration of 0.6 mM	Arora et al., 2016
92.	*Bacillus megaterium* PC-6	4-chloro-2-nitrophenol	Decolorized up to a concentration of 1.5 mM	Arora et al., 2016
93.	*Bacillus aryabhattai* PC-7	4-chloro-2-nitrophenol	Decolorized up to concentration of 2.0 mM	Arora et al., 2016
94.	*Bacillus aerophilus* PC-8	4-chloro-2-nitrophenol	Decolorized up to concentration of 0.6 mM	Arora et al., 2016
95.	*Bacillus siamensis* PC-9	4-chloro-2-nitrophenol	Decolorized up to concentration of 0.8 mM	Arora et al., 2016
96.	*Bacillus amyloliquefaciens* PC-10	4-chloro-2-nitrophenol	Decolorized up to concentration of 0.9 mM	Arora et al., 2016
97.	*Bacillus subtilis* MF447840	4-chlorophenol	Degraded 4-chlorophenol up to of 1,000 mg/L	Sandhibigraha et al., 2020
98.	*Bacillus cereus* PU	Trinitrophenol	Used trinitrophenol as nitrogen source and degraded via Hydride-Meisenheimer complex.	Singh et al., 2011
**D. Biodegradation of Polyaromatic hydrocarbons**				
99.	*Bacillus subtilis* 3KP	Naphthalene and Phenanthrene	Metabolized via hydroxy-2-naphthoic acid, salicylic acid, and pyrocatechol	Ni'matuzahroh et al., 2017
100.	*Bacillus fusiformis*	Naphthalene	Degraded via o-phthalic acid and benzoic acid,	Lin et al., 2010
101.	*Bacillus cereus* RKS4	Naphthalene	Catechol and 2-naphthol were identified as major metabolites of naphthalene degradation.	Sonwani et al., 2019
102.	*Bacillus* sp. SBER3	Anthracene and Naphthalene	Degraded 83.4% of anthracene and 75.1% of and naphthalene in 6 days.	Bisht et al., 2014
103.	*Bacillus subtilis* DM-04	Pyrene	Used it as its carbon source and energy	Das and Mukherjee, 2007
104.	*Bacillus subtilis* BM-1	Fluorene	Degrade 86% of 50 mg/L fluorine with 21 days	Salam and Obayori, 2014
105.	*Bacillus amyloliquefaciens* BR1	Fluorene	Degrade 82% of 50 mg/L fluorine with 21 days	Salam and Obayori, 2014
106.	*Bacillus subtilis* BTM4i	Benza-pyrene	Utilized as a sole source of carbon and energy and degradation ability was chromosomally coded.	Lily et al., 2010
107.	*Bacillus pumilus* (MTCC 1002)	Pyrene	Co-metabolize 64% of 50 μg/ml pyrene via 9-methoxyphenanthrene and phthalate	Khanna et al., 2011
**E. Biotransformation and detoxification of heavy metals**				
108.	*Bacillus* sp. strain FM1	Chromium	Completely reduced 100 mg/L Cr(VI) within 48 h	Masood and Malik, 2011
109.	*Bacillus* sp. strain KSUCr9a	Chromium	rapidly reduce up to 100 μM of Chromium within 24 h	Ibrahim et al., 2012
110.	*Bacillus sphaericus* AND 303	Chromium	300 μM Cr(VI) reduction by cell extracts (4.56 mg protein/mL) of strain AND303	Pal et al., 2005
111.	*Bacillus* sp. FY1	Chromium	Reduced 78–85% of Cr(VI) (100–200 mg/l) within 24 h	Xiao et al., 2017
112.	*Bacillus* sp. MNU16	Chromium	Reduced 75% of Cr(VI) of 50 mg/L within 72 h.	Upadhyay et al., 2017
113.	*Bacillus amyloliquefaciens* CSB 9	Chromium	Reduced Cr(VI) to Cr (III) that was immobilized to the bacterial cell surface and subsequent intercellular accumulation of Cr (III) along with the formation of coagulated cell precipitate	Das et al., 2014
114.	*Bacillus cereus* S612	Chromium	Reduced chromate under aerobic conditions	Wang et al., 2015
115.	*Bacillus cereus*	Chromium	Reduced Cr(VI) to Cr (III). Cr(III) precipitates were accumulated on bacterial surfaces	Chen et al., 2012c
116.	*Bacillus* sp. ES 29	Chromium	A copper (Cu2+) stimulated soluble Cr(VI)-reducing enzyme reduced Cr(VI) to Cr (III)	Camargo et al., 2003
117.	*Bacillus* sp. MH778713	Chromium	Accumulated up to 100 mg Cr(VI)/g of cells and tolerate up to 15,000 mg/L Cr (VI)	Ramírez et al., 2019
118.	*Bacillus cereus* TN10	Chromium	Detected chromate transporters in the genome	Hossain et al., 2020
119.	*Bacillus cereus* 12-2	Lead	Transformed Pb(II) into nanosized rod-shaped Ca2.5Pb7.5(OH)2(PO4)6 crystal	Chen et al., 2016
120.	*Bacillus* sp. KK-1	Lead	Converted Pb(NO3)2 into lead sulfide (PbS) and lead silicon oxide (PbSiO3)	Govarthanan et al., 2013
121.	*Bacillus* cereus BPS-9	Lead	Bioaccumulation of lead by biosorption	Sharma and Shukla, 2021
122.	*Bacillus megaterium*	Selenium	Reduced Se(IV) to red element Se (III)	Mishra et al., 2011
123.	*Bacillus subtilis*	Selenium	Proposed physiological mechanisms regulating the selenite reduction	Garbisu et al., 1995
124.	*Bacillus selenatarsenatis* SF-1	Selenium	Reduced selenate to selenite through anaerobic respiration, and subsequently into elemental selenium	Kuroda et al., 2015
125.	*Bacillus selenitireducens* MLS10	Selenium	Enzyme respiratory selenite [Se(IV)] reductase (Srr) was characterized.	Wells et al., 2019
126.	*Bacillus cereus* CM100B	Selenium	Produced selenium nanoparticles by transformation of toxic selenite (SeO32-) anions into red elemental selenium (Se0) under aerobic conditions	Dhanjal and Cameotra, 2010
127.	*Bacillus mycoides* strain SeITE01	Selenium	Reduced selenite (SeO32-) anions into red elemental selenium (Se0) with the formation of selenium nanoparticles.	Lampis et al., 2014
128.	*Bacillus thuringiensis*	Uranium	Transformation from U(VI) into nano-uramphite	Pan et al., 2015
129.	*Bacillus licheniformis* SPB-2	Copper	Reduced [Co(III)–EDTA]– to [Co(II)–EDTA]2– which was further absorbed by strain SPG-2	Paraneeiswaran et al., 2015
130.	*Bacillus firmus* strain TE7	Chromium and Arsenic	Reduced Cr(VI) to Cr (III) and oxidized As(III) to As(V)	Bachate et al., 2013
131.	*Bacillus* sp. strain Arzi	Mb	Reduced molybdate to molybdenum blue	Othman et al., 2013
132.	*Bacillus thuringiensis* OSM29	Ni and Cu	Biosorption capacity of the strain OSM29 for the metallic ions was highest for Ni (94%) which was followed by Cu (91.8%).	Oves et al., 2013
**F. Degradation of Natural Compounds**				
133.	*Bacillus macerans* JJ-lb	Protocatechuate	Completely mineralized	Crawford et al., 1979
134.	*Bacillus* sp.	3-Hydroxybenzoate	Completely mineralized via protocatechuate	Mashetty et al., 1996
135.	*Bacillus brevis* PHB-2	4-Hydroxybenzoate	Completely mineralized via protocatechuate	Crawford, 1976
136.	*Bacillus circulans* strain 3	4-Hydroxybenzoate	Completely mineralized via protocatechuate	Crawford, 1976
137.	*Bacillus laterosporus*PHB-7a	4-Hydroxybenzoate	Completely mineralized via gentisate	Crawford, 1976
138.	*Bacillus* sp. B-1	Cinnamic acid	Degraded via benzoic acid	Peng et al., 2003
139.	*Bacillus* sp. B-1	4-Coumaric acid	Degraded via 4-hydroxybenzoic acid	Peng et al., 2003
140	*Bacillus* sp. B-1	Ferculic acid	Metabolized via 4-hydroxy-3-methoxyphenyl-beta-hydroxypropionic acid, vanillin, and vanillic	Peng et al., 2003
141.	*Bacillus pumilus* W1	Cholesterol degradation	Cholesterol as only carbon and energy	Wali et al., 2019
142.	*Bacillus ligniniphilus* L1	Alkaline lignin	Degraded via three different pathway including gentisate pathway, benzoic acid pathway, and the β-ketoadipate pathway	Zhu et al., 2017
**G. Degradation of Explosives**				
143.	*Bacillus* sp. J8A2	Pentaerythritol tetranitrate	Utilized it as a nitrogen source	Yerson and Christian, 2013
144.	*Bacillus* sp. SF	Trinitrotoluene	Transformed to hydroxylaminodinitrotoluene, 4-amino-2,6-dinitrotoluene; 2-amino-4,6-dinitrotoluene, different azoxy compounds, 2,6-diaminonitrotoluene and 2,4-diaminonitrotoluene.	Nyanhongo et al., 2008
145.	*Bacillus cereus*	Trinitrotoluene	Transformed into 2,4-dinitrotoluene and 4-aminodinitrotoluene derivates,	Mercimek et al., 2013
146.	*Bacillus* sp. ATCC51912	Propylene glycol dinitrate	Sequentially denitrated to propylene glycol mononitrate and propylene glycol	Sun et al., 1996
147.	*Bacillus* sp. ATCC51912	Glycerol trinitrates	Sequential denitration of glycerol trinitrates to glycerol via glycerol dinitrate isomers and glycerol mononitrate isomers	Meng et al., 1995
**H. Degradation of Drugs**				
148.	*Bacillus thuringiensis* B1	Naproxen	Degraded via salicylic acid and catechol	Górny et al., 2019
149.	*Bacillus thuringiensis* B1	Ibuprofen	Degraded it via hydroxyibuprofen	Marchlewicz et al., 2017
150.	*Bacillus drentensis* S1	Acetaminophen	Degraded via 2-isopropyl-5-methylcyclohexanone and phenothiazine	Chopra and Kumar, 2020
**I. Degradation of other Xenobiotic compounds**				
151.	*Bacillus salamalaya* 139SI	Crude oil waste	Degraded 88% of the total petroleum hydrocarbons within 42 days in mineral media containing 1% of crude oil waste.	Ismail and Dadrasnia, 2015
152.	*Bacillus* sp. BCBT21	Plastic bags	Produced extracellular hydrolase enzymes including lipase, carboxymethyl cellulase, xylanase, chitinase, and protease	Dang et al., 2018
153.	*Bacillus pumilus*B12	Poly-lactic acid	Degraded plylacitc acid film within 48-h by the release of L-lactate monomers	Bonifer et al., 2019
154.	*Bacillus* sp. strain 4	Pyridine	Used it sole C, N and energy source	Watson and Cain, 1975

### *Bacilli*-Mediated Degradation of 4-Chloro-2-Nitrophenol

4-Chloro-2-nitrophenol is a chloro derivative of nitrophenol that is widely used for the synthesis of dyes, pesticides, drugs, and chemicals (Arora et al., 2018). Due to its wide range of applications, this compound has been detected in a variety of sources including industrial effluents. It is highly toxic to living beings and may cause methemoglobinemia in human beings. So far, several physicochemical and biological methods have been used for the 4-chloro-2-nitrophenol degradation (Bruhn et al., 1988; Beunink and Rehm, 1990; Saritha et al., 2007; Gharbani et al., 2010; Hashemi et al., 2017; Arora et al., 2018). In this sub-section, the role of *Bacillus* species in the 4-chloro-2-nitrophenol degradation is summarized.

**Figure 1:**
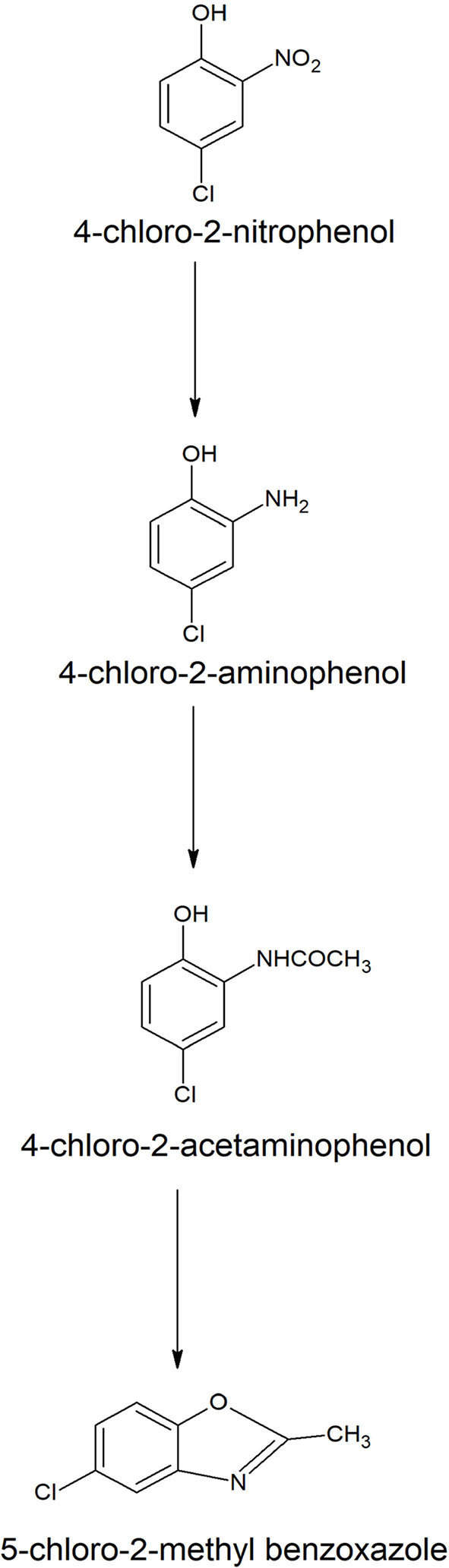
Biotransformation pathway of 4-chloro-2-nitrophenol in *Bacillus* spp. (Arora, 2012).

Many *Bacilli* have been characterized for their ability to decolorize the yellow color of 4-chloro-2-nitrophenol in the presence of additional carbon source (Arora et al., 2018). A marine bacterium, *Bacillus* sp. MW-1 (Arora and Jain, 2012), and a soil bacterium, *Bacillus subtilis* RKJ 700 (Arora, 2012) decolorized and transformed 4-chloro-4-nitrophenol into 5-chloro-2-methylbenzoxazole via detoxification mechanism. In this mechanism, 4-chloro-2-nitrophenol initially reduced to 4-chloro-2-aminophenol, which is further acetylated to 4-chloro-2-acetaminophenol. The next step involves the conversion of 4-chloro-2-acetaminophenol to 5-chloro-2-methylbenzoxazole (Figure 1). Recently, ten bacterial strains belonging to *Bacillus* isolated from a wastewater sample showed decolorization of 4-chloro-2-nitrophenol in the presence of glucose. One of a bacterium, identified as *Bacillus aryabhattai* strain PC-7 decolorized 4-chloro-2-nitrophenol up to a concentration of 2.0 mM and transformed it into 5-chloro-2-methylbenzoxazole (Arora et al., 2016).

Besides *Bacillus* spp., several other bacteria are also capable of transforming 4-chloro-2-nitrophenol to 5-chloro-2-methylbenzoxazole. These bacteria belong to the genera *Pseudomonas, Leuconostoc*, and *Paenibacillus* (Arora et al., 2016). The memberes of genus *Bacillus* were unable to completely mineralize 4-chloro-2-nitrophenol, but they transformed 4-chloro-2-nitrophenol via a detoxification mechanism. The complete degradation of 4-chloro-2-nitrophenol was studied using an *Exiguobacterium* sp. PMA (Arora et al., 2012), a co-culture of *Enterobacter* cloacae and an *Alcaligenes* sp. TK-2 (Beunink and Rehm, 1990), and the genetically engineered bacterium, *Pseudomonas* sp. N31 (Bruhn et al., 1988).

### *Bacilli-*Mediated Degradation of Polycyclic Aromatic Hydrocarbons

Polycyclic aromatic hydrocarbons (PAHs) are those aromatic compounds which contain two or more fused aromatic rings in linear, angular, or cluster arrangements (Masih and Taneja, 2006). Examples are naphthalene, anthracene, fluorene, phenanthrene, fluoranthene, pyrene, and benzo[a]pyrene (Abdel-Shafy and Mansour, 2016). PAHs are toxic to the living world and some of them are considered as possible carcinogens. Therefore, the USEPA has listed 16 PAHS in its priority list of pollutants (Zelinkova and Wenzl, 2015). Major sources of PAHs pollution include fuel combustion, automobiles, spillage of petroleum products, waste incinerators, and industrial effluents (Abdel-Shafy and Mansour, 2016). In this section, *the Bacilli*-mediated degradation of a few PAHs is summarized.

**Figure 2:**
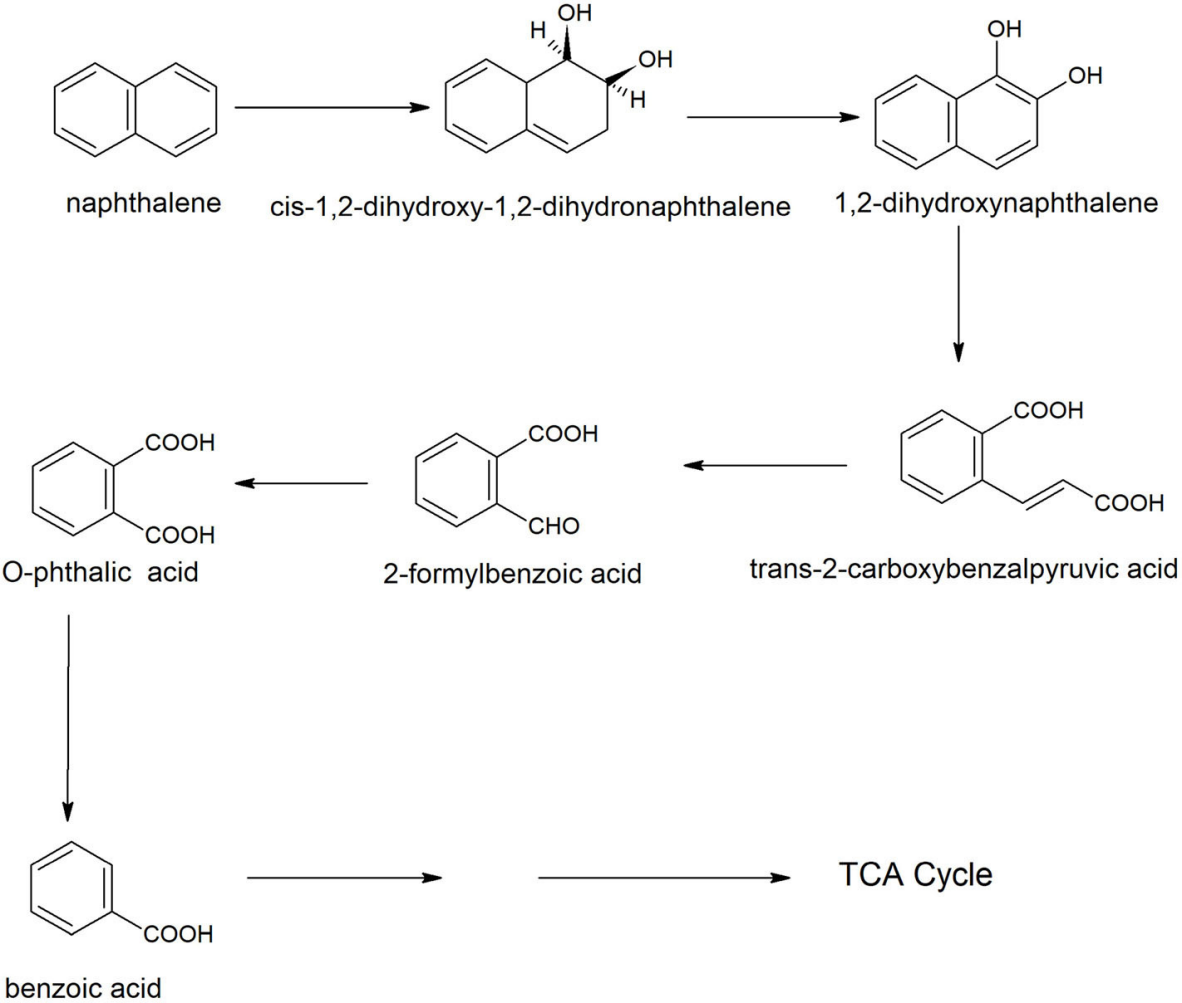
A degradation pathway of naphthalene in *Bacillus fusiformis* strain BFN (Lin et al., 2010).

Naphthalene is the simplest example of polycyclic aromatic compounds. An early study on naphthalene degradation by *B. cereus* ATCC14579 showed the complete transformation of naphthalene to 1-naphthol (Cerniglia et al., 1984). A possible degradation pathway of naphthalene was studied in *B. fusiformis* BFN that was isolated from oil refining wastewater sludge (Lin et al., 2010). The naphthalene degradation was initiated with 1, 2-dioxygenation, resulting in the formation of *cis*-1,2-dihydroxy-1,2-dihydronaphthalene that dehydrogenated to 1,2-dihydroxynaphthalene. The *ortho-*ring cleavage of 1,2-dihydroxynaphthalene produced *o*-phthalic acid via the formation of trans-2-carboxybenzalpyruvic acid and 2-formyl benzoic acid (Figure 2). The phthalic acid decarboxylated to benzoic acid that further metabolized carbon dioxide and water. Ni'matuzahroh et al. (2017) studied the degradation of naphthalene and phenanthrene by *B. subtilis* 3KP that degraded them via the formation of 1-hydroxy-2-naphthoic acid, salicylic acid, and pyrocatechol. Sonwani et al. (2019) reported that *B. cereus* RKS4 degraded naphthalene via the formation of 2-naphthol and catechol. Annweiler et al. (2000) studied the degradation of naphthalene in *B. thermoleovorans* Hamburg 2 under thermophilic conditions (60° C). *B. thermoleovorans* Hamburg 2 utilized naphthalene as the sole source of carbon and energy and degraded it via formation of 1-naphthol, 2-naphthol, 2,3-dihydroxynaphthalene, 2-carboxycinnamic acid, phthalic acid, and benzoic acid, coumarin, 3-(2-Hydroxyphenyl)-propanoic acid, 2,3-dihydrocoumarin, 2-hydroxybenzoic acid (salicylic acid) and 2-carboxycinnamic acid.

**Figure 3:**
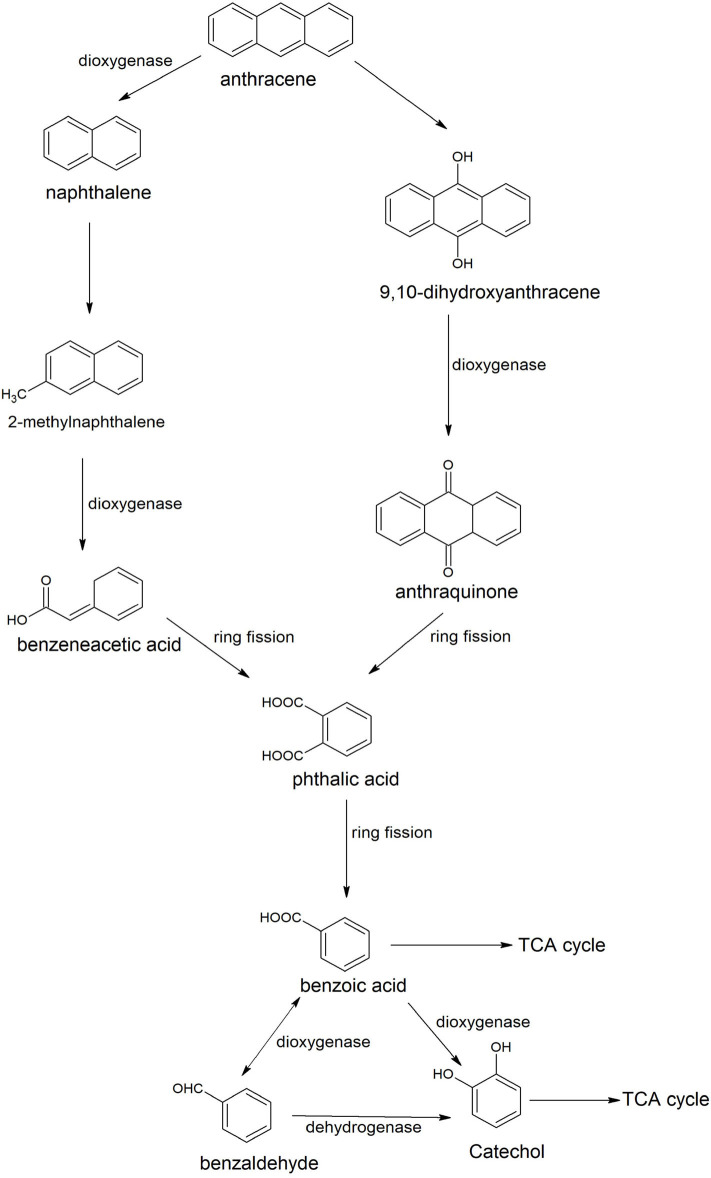
Degradation pathways of anthracene by *Bacillus licheniformis* MTCC 5514 (Swaathy et al., 2014).

Anthracene is an integral part of many carcinogenic PAHs; therefore it has been detected easily in several contaminated sites of PAHs. Many *Bacilli* have been identified and characterized for degradation of anthracene. Examples are *Bacillus* sp. SBER3 (Bisht et al., 2014), *B. cereus* JMG-01 (Das et al., 2017), *B. licheniformis* MTCC 5514 (Swaathy et al., 2014), *B. cereus* S13 (Bibi et al., 2018), and *B*. *badius* D1 (Sarwade and Gawai, 2014). Das et al. (2017) studied the degradation pathway of anthracene for *B. cereus* JMG-01 that degraded 98% of 500 ppm anthracene. The anthracene degradation was initiated with the formation of naphthalene and naphthalene-2-methyl. In the next step, a dioxygenase enzyme catalyzed oxidation of naphthalene-2-methyl to benzene acetic acid. Further, benzene acetic acid underwent ring cleavage to produce phthalic acid and benzaldehyde. Benzaldehyde converted to catechol that degraded via either *ortho* or *meta* ring cleavage. Swaathy et al. (2014) reported the existence of two degradation pathways in biosurfactant mediated biodegradation of anthracene by *B. licheniformis* (MTCC 5514). One pathway proceeded with the formation of naphthalene, naphthalene 2-methyl, phthalic acid, and benzene acetic acid. Another pathway was initiated with dioxygenation of anthracene to produce di-hydroxy anthracene, which, further transformed to anthraquinone by a dioxygenase enzyme (Figure 3). Anthraquinone was further degraded with the formation of phthalic acid, benzaldehyde or benzoic acid, and catechol. Metabolites of both of the pathways (9, 10-dihydroxyanthracene, anthraquinone, benzene acetic acid, and catechol) were also reported in the anthracene degradation pathway of *B. cereus* S13 that utilized it as the sole source of carbon and energy (Bibi et al., 2018). Another pathway of anthracene was studied in an alkaliphilic bacterium *B. badius* D1 that was able to degrade anthracene at a concentration of 50 mg/100 ml at pH 9.0 (Sarwade and Gawai, 2014). In this pathway, anthracene was initially oxidized to 1, 2-dihydoxyanthracene that further oxidized (3Z)-4-[3-hydroxy (2-naphthyil)-2-oxobut-3-enoic acid with subsequent conversion to 2-hydroxynaphthoic acid, Further oxidation resulted in the formation of phthalic acid that was degraded via formation of simple aliphatic compounds.

### *Bacilli*-Mediated Degradation of Pyrethroid Insecticides

Pyrethroid insecticides are synthetic pyrethroids which are analogs to natural pyrethrins extracted from Chrysanthemum cinerariaefolium (Cycoń and Piotrowska-Seget, 2016). Representative compounds of these pesticides are cyhalothrin, fenpropathrin, deltamethrin, cypermethrin, cyfluthrin, and bifenthrin (Zhan et al., 2020). They are used to control a broad spectrum of pests in households and agriculture fields. Due to their wide range of applications in agriculture fields, they have been spread into soil and water and create environmental problems because of their toxic nature (Zhan et al., 2020). Many *Bacilli* have been isolated and characterized for the degradation of several pyrethroids (Chen et al., 2012b; Cycoń and Piotrowska-Seget, 2016; Bhatt et al., 2020). In this section, *Bacilli*-mediated degradation of various pyrethroids is discussed.

**Figure 4:**
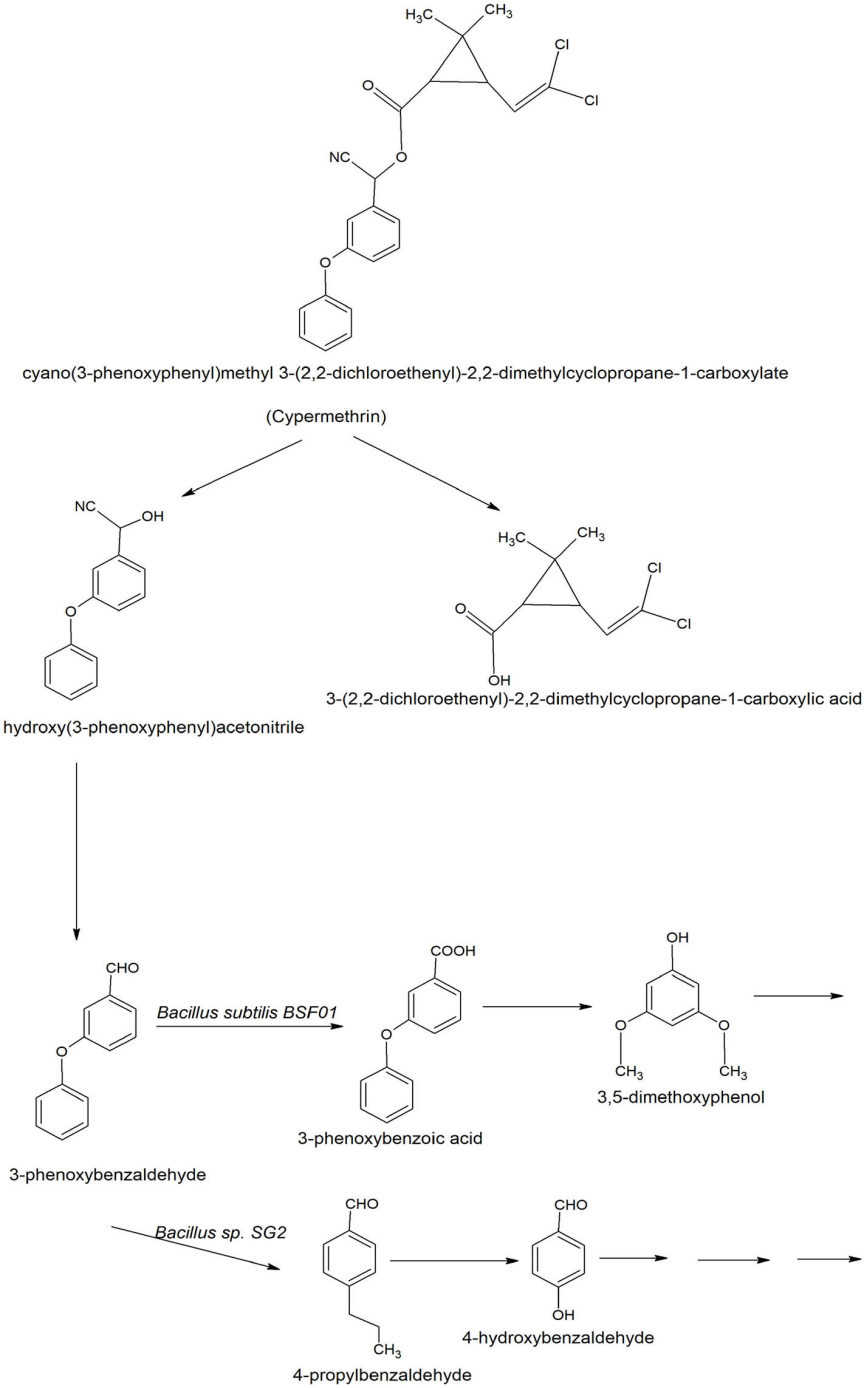
Degradation pathways of cypermethrin in *Bacillus* sp. SG2 and *Bacillus subtilis* BSF01 (Xiao et al., 2015; Pankaj et al., 2016).

**Figure 5:**
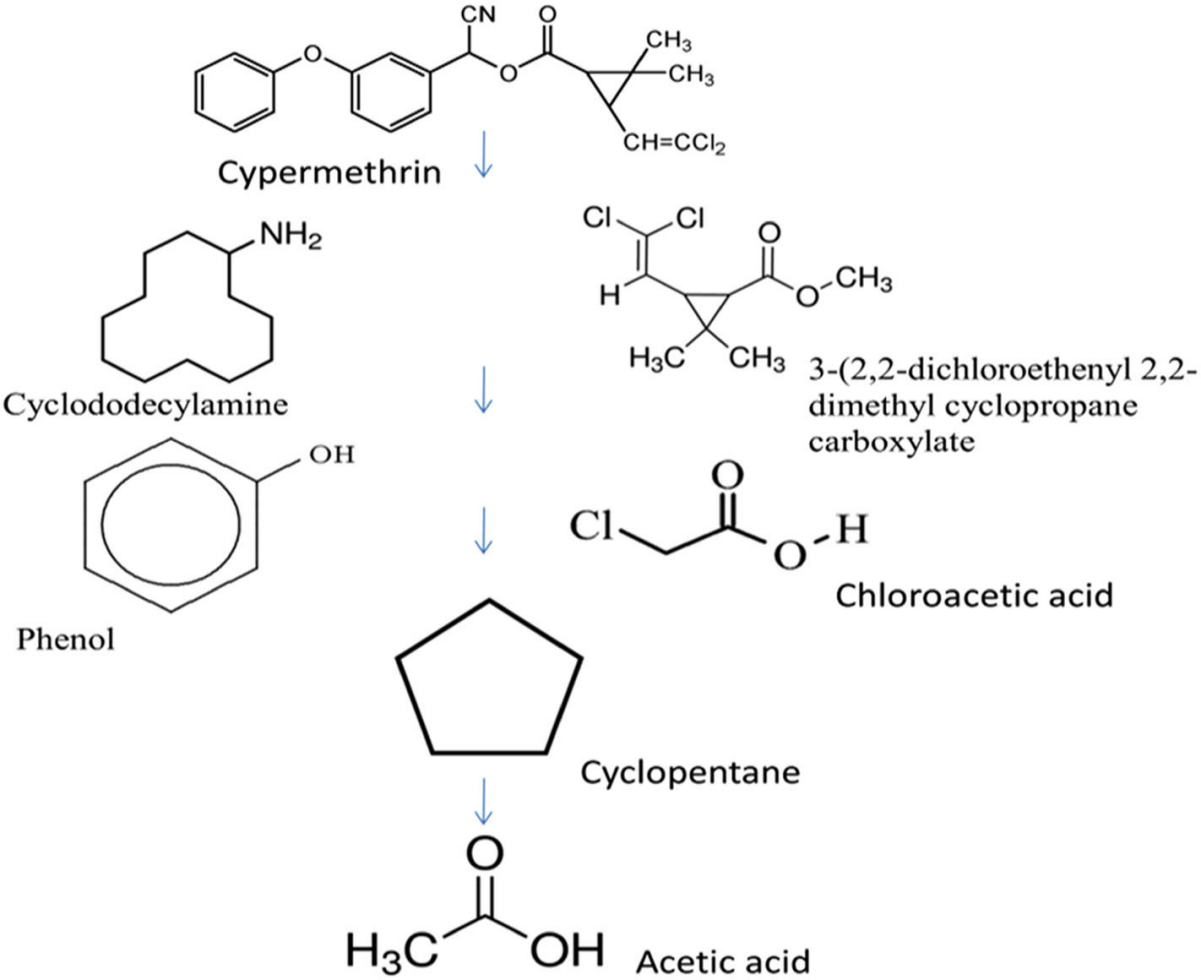
Degradation pathways of cypermethrin in *Bacillus subtilis* strain 1D (adapted from Gangola et al., 2018).

The degradation of cypermethrin is well-studied in some *Bacilli* including *Bacillus* sp. SG2 (Pankaj et al., 2016), *B. subtilis* BSF01 (Xiao et al., 2015), *B. subtilis* strain 1D (Gangola et al., 2018), *Bacillus* sp. AKD1 (Tiwary and Dubey, 2016), *Bacillus* sp. ISTDS2 (Sundaram et al., 2013) and *B. licheniformis* B-1 (Lai et al., 2012). The initial steps of degradation pathways of cypermethrin are common in *Bacillus* sp. SG2 and *B. subtilis* BSF01 (Xiao et al., 2015; Pankaj et al., 2016). Cypermethrin was initially transformed into two metabolites: α-hydroxy-3-phenoxy-benzene acetonitrile and 3-(2,2-dichloroethenyl)-2,2-dimethyl cyclopropanecarboxylate). The unstable compound, α-hydroxy-3-phenoxy-benzene acetonitrile was spontaneously transformed into 3-phenoxybenzaldehyde (Figure 4). Further degradation of 3-phenoxybenzaldehyde proceeded via a different route in *Bacillus* sp. SG2 and *B. subtilis* BSF01. In *B*. *subtilis* BSF01, the degradation of 3-phenoxybenzaldehyde proceeded via the formation of 3-phenoxybenzoic acid and 3, 5-dimethoxyphenol (Xiao et al., 2015). However, in *Bacillus* sp. SG2, 3-phenoxybenzaldehyde was further converted to 4-propylbenzaldehyde and then to 4-hydroxybenzoate that was transformed to phenyl ester of *o*-phenoxy benzoic acid (Pankaj et al., 2016). The phenyl ester of *o*-phenoxy benzoic acid was degraded via the formation of phenol-M-tert-butyl, phenol, and aliphatic hydrocarbons or short-chain compounds. Another pathway of degradation of cypermethrin was studied in *B. subtilis* strain 1D (Gangola et al., 2018). In this pathway, cypermethrin was initially transformed into 3-(2, 2-dichloro ethenyl)-2,2-dimethyl-cyclopropanecarboxylate and cyclododecylamine due to hydrolysis of the ester linkage (Figure 5). The unstable compound, cyclododecylamine oxidized to phenol which reacted with water to form cyclopentane that transformed into aliphatic compounds like acetic acid and decanoic acid. Another metabolite, 3-(2, 2-dichloro ethenyl)-2,2-dimethyl-cyclopropanecarboxylate was hydrolyzed to form chloroacetic acid (Gangola et al., 2018).

**Figure 6:**
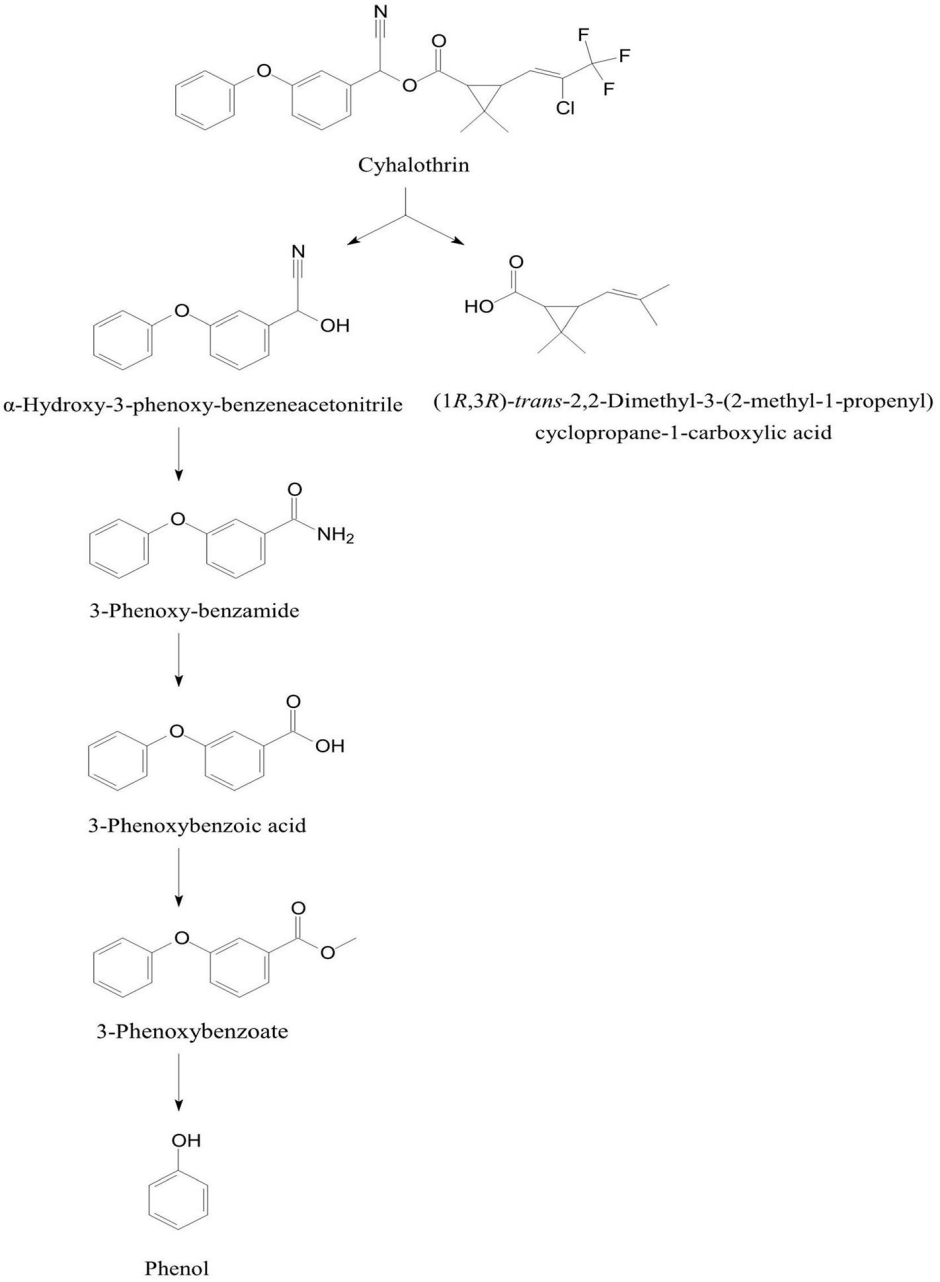
Degradation pathway of cyhalothrin in *Bacillus thuringiensis* ZS-19 (adapted from Chen et al., 2015).

The degradation pathway of cyhalothrin [(RS)-α-Cyano-3-phenoxybenzyl-(Z)-(1RS,3RS)-(2-chloro-3,3, 3-trifluoro propenyl)-2,2-dimethylcyclopropanecarboxylate)] was studied in *B. thuringiensis* ZS-19 that initiated degradation of cyhalothrin by cleavage of the carboxyl ester linkage through hydrolysis to form α-hydroxy-3-phenoxy-benzeneacetonitrile and (1RS,3RS)*-trans*-2,2-dimethyl-(2-methyl-1-propenyl)cyclopropane-1-carboxylic acid (Chen et al., 2015). The α-hydroxy-3-phenoxy-benzeneacetonitrile was converted to 3-phenoxybenzote acid via 3-phenoxyphenyl acetonitrile, N-(2-isoproxy-phenyl)-4-phenoxy-benzamide, and 3-phenoxybenzaldehyde (Figure 6). Further degradation of 3-phenoxybenzoate was proceeded through cleavage of diaryl bond to produce and phenol that was degraded via aromatic ring cleavage (Chen et al., 2015).

**Figure 7:**
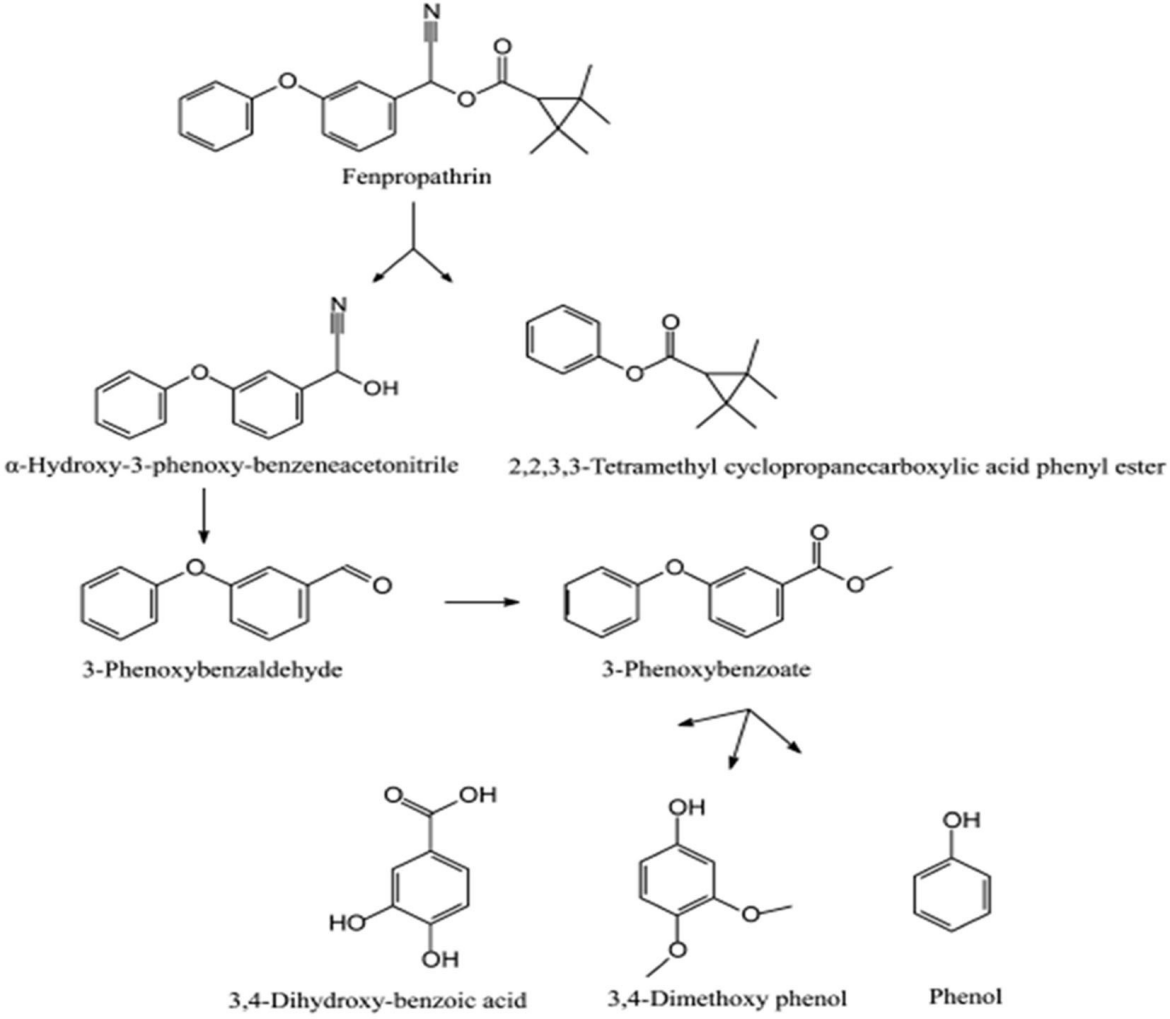
Degradation pathway of fenpropathrin in *Bacillus* sp. DG-02 (Reprinted (adapted) from Chen et al., 2014). Copyright (2014) American Chemical Society.

The degradation pathway of fenpropathrin(α-cyano-3-phenoxybenzyl 2,2,3,3-tetramethylcyclopropanecarboxylate) was studied in *Bacillus* sp. DG-02, isolated from a soil sample collected from the aerobic pyrethroid-manufacturing wastewater treatment system of China (Chen et al., 2014). Initially, fenpropathrin was converted to α-hydroxy-3-phenoxybenzeneacetonitrile and 2, 2, 3, 3-tetramethylcyclopropanecarboxylic acid phenyl ester due to cleavage of the carboxyl ester linkage (Figure 7). In the next step, unstable compound α-hydroxy-3-phenoxybenzeneacetonitrile was spontaneously transformed into 3-phenoxybenzaldehyde, which oxidized to 3-phenoxybenzoate. Subsequent degradation of 3-phenoxybenzoate produced 3, 4-dihydroxybenzoic acid, 3, 4-dimethoxyphenol, and phenol (Chen et al., 2014).

### *Bacilli-*Mediated Degradation of Organophosphorus Pesticides

Organophosphorus pesticides are a large group of chemicals that widely used for protecting crops, livestock from various pests (Sidhu G. K. et al., 2019). Commonly used organophosphates are malathion, parathion, methyl parathion, chlorpyrifos, diazinon, fenitrothion, dichlorvos, ethion, and monocrotophos (Sidhu G. K. et al., 2019). These compounds act as an inhibitor of an acetylcholinesterase enzyme that hydrolyzes the neurotransmitter acetylcholine found in both the peripheral and central nervous systems (Robb and Baker, 2020). This inhibition mechanism involves the phosphorylation of the serine hydroxyl group present on the active site of acetylcholinesterase (Robb and Baker, 2020). In this section, the role of *Bacilli* for the degradation of organophosphorus pesticides is discussed.

Many reports have been published dealing with the potential applications of *Bacilli* to degrade organophosphorus pesticides. Bhadbhade et al. (2002) reported mineralization of monocrotophos to carbon dioxide, ammonia, and phosphates by *B*. *megaterium* MCM B-423, isolated from soil exposed to monocrotophos. The enzymes, phosphatase, and esterase were involved in the monocrotophos degradation pathway, which proceeds via acetic acid, methylamine, and one unidentified metabolite. Dash and Osborne (2020) studied degradation pathways of monocrotophos by *B. aryabhattai* strain VITNNDJ5 in artificially contaminated soil and reported that *B. aryabhattai* may be degraded monocrotophos via three routes; one route proceeds with the hydrolysis of monocrotophos into dimethyl phosphate that was degraded further into phosphoric acid and acetic acid esters by hydrolase and monooxygenase enzymes. The second degradation pathway was initiated with the demethylation of monocrotophos to N-(hydroxymethyl) acetamide that was further degraded into acetamide. Acetamide converted into acetic that entered the TCA cycle. In the third route, monocrotophos, monocrotophos converted into orthophosphoric acid and acetic acid via formation of phosphonoacetate intermediate.

Another *Bacillus* sp. TAP-1 that was isolated from sewage sludge of a wastewater treating system of organophosphorus pesticide was capable of hydrolyzing high concentrations of triazophos (50–400 mg/l) (Tang and You, 2012). Salunkhe et al. (2013) reported the biodegradation of an organophosphorus insecticide, profenofos by four *B. subtilis* strains, namely, DR-39, CS-126, TL-171, and TS-204, isolated from grapevines or grape rhizosphere and 4-bromo-2-chlorophenol was identified as a metabolite. A marine *Bacillus* sp. strain C5 isolated from the China Bohai Sea produced an extracellular esterase that hydrolyzed methyl parathion to 4-nitrophenol and other metabolites (Hao et al., 2014). Anwar et al. (2009) reported that *B. pumilus* C2A1 isolated from a soil sample collected from the cotton field, degraded chlorpyrifos, and its first hydrolysis metabolite 3,5,6-trichloro-2-pyridinol. Strain C2A1 degraded maximum amounts of chlorpyrifos at alkaline pH (8.5) and high inoculums bacterial density. Pailan et al. (2015) isolated organophosphates-degrading bacterium, *B*. aryabhattai strain SanPS1 from a soil sample of an agricultural field located at Narigram in Burdwan district of West Bengal, India. Strain SanPS1 degraded parathion via the formation of 4-nitrophenol and 4-nitrocatechol.

### *Bacilli-*Mediated Degradation of Organochlorine Pesticides

Organochlorine pesticides are a group of chlorinated compounds, which include DDT, methoxychlor, endosulfan, dieldrin, chlordane, toxaphene, mirex, kepone, lindane, and benzene hexachloride (Jayaraj et al., 2016). These compounds are widely distributed to the environment due to applications. In this section, *Bacilli*-mediated degradation of organochlorine pesticides is discussed.

*B. subtilis* MTCC 8561 utilized endosulfan and endosulfan sulfate as its sulfur sources and degraded both of them via the formation of endosulfan diol and endosulfan lactone (Kumar et al., 2014). Awasthi et al. (2003) also reported the degradation of alpha and beta isomers of endosulfan via the formation of endosulfan diol and endosulfan lactone using the co-culture of *Bacillus* sp. MTCC 4444 and *Bacillus* sp. MTCC 4445. Seralathan et al. (2014) postulated the role of cytochrome P450 BM3 of *B. megaterium* in biotransformation of endosulfan through *in silico* prediction approach. Kumar and Philip (2006) reported that the anaerobic degradation of endosulfan, endosulfan ether, and endosulfan lactone using mixed bacterial culture containing two strains of *B*. *circulans* and one strain of *Staphylococcus* sp. All three strains metabolized endosulfan via hydrolysis pathway with the formation of carbenium ions and/or ethylcarboxylates, which further converted into simple hydrocarbons (Kumar and Philip, 2006).

### *Bacilli*-Mediated Degradation of Herbicides

Herbicides are chemical substances that are generally used to control the growth of unwanted plants (Herrera-Herrera et al., 2016). These are known as weed killers and divided into two categories: contact herbicides and systematic herbicides. Contact herbicides are localized in action and affect only the part of the plant that they touch (Herrera-Herrera et al., 2016). Examples are diclofop, dinoseb, diquat, and paraquat (Herrera-Herrera et al., 2016). Systemic herbicides may be translocated to other parts of the plants. Examples are atrazine, quinclorac, glyphosate 2,4-dichlorophenoxyacetic acid (2,4-D), and simazine (Herrera-Herrera et al., 2016). In this section, the role of *Bacilli* for the degradation of herbicides is discussed.

*Bacillus subtilis* HB-6 isolated from industrial wastewater utilized atrazine as its sole nitrogen source for growth and mineralized it via formation of hydroxyatrazine, cyanuric acid, and urea (Wang et al., 2014). The atrazine-degrading genes, *trzN, atzB*, and *atzC* which encode the enzymes to converting atrazine to cyanuric acid were detected in strain HB-6 (Wang et al., 2014). Liu et al. (2014) studied the degradation of a highly selective auxin herbicide, quinclorac (3,7-dichloro-8-quinoline-carboxylic) by *B. megaterium* Q3 isolated from the root of tobacco grown in quinclorac contaminated soil. Strain Q3 transformed quinclorac to 3, 7-dichloro-8-methyl-quinoline, 3-chlorin-8-quinoline-carboxylic and 8-quinoline-carboxylic (Liu et al., 2014).

### *Bacilli*-Mediated Degradation of Drugs

Ibuprofen and naproxen are known as non-steroidal anti-inflammatory drugs and widely used to control mild to moderate pain, fever, inflammation, menstrual cramps, and types of arthritis (Marchlewicz et al., 2017). Due to the high consumption of these drugs, they have been detected in the effluents of several biological wastewater treatment systems as environmental pollutants (Marchlewicz et al., 2017). In this section, the *Bacillus*-medited degradation of ibuprofen and naproxen is discussed.

To date, only one species of *Bacillus*, i.e., *B. thuringiensis* B1 was able to degrade both Ibuprofen and naproxen (Marchlewicz et al., 2017; Górny et al., 2019). The effective degradation of both of these drugs occurred in the presence of glucose. *B. thuringiensis* B1 was able to degrade ibuprofen and naproxen up to concentrations of 25 mg/Land 12 mg/L, respectively.

**Figure 8:**
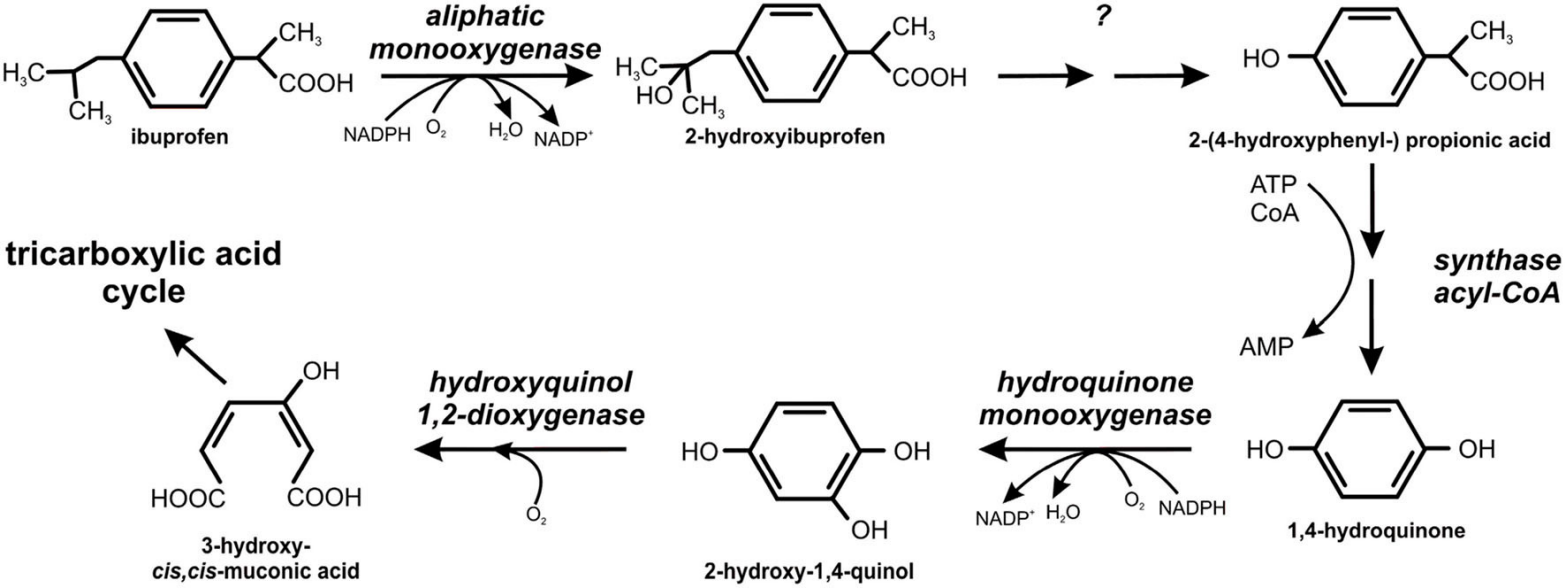
Degradation pathway of ibuprofen in *Bacillus thuringiensis* B1 (adapted from Marchlewicz et al., 2017).

The degradation pathways of ibuprofen and naproxen were studied in *B. thuringiensis* B1. The first step of the ibuprofen degradation is hydroxylation of ibuprofen into 2-hydroxyibuprofen by aliphatic monooxygenase (Marchlewicz et al., 2017). The second step was the conversion of 2-hydroxyibuprofen to 2-(4-hydroxyphenyl-) propionic acid that was further transformed into 1,4-hydroquinone by acyl-CoA synthase/thiolase activity (Figure 8). In the next step, a hydroquinone monooxygenase catalyzed conversion of 1,4-hydroquinone to 2-hydroxy-1,4-quinol which cleaved to 3-hydroxy-*cis, cis*-muconic acid by hydroxyquinol 1,2-dioxygenase (Marchlewicz et al., 2017).

**Figure 9:**
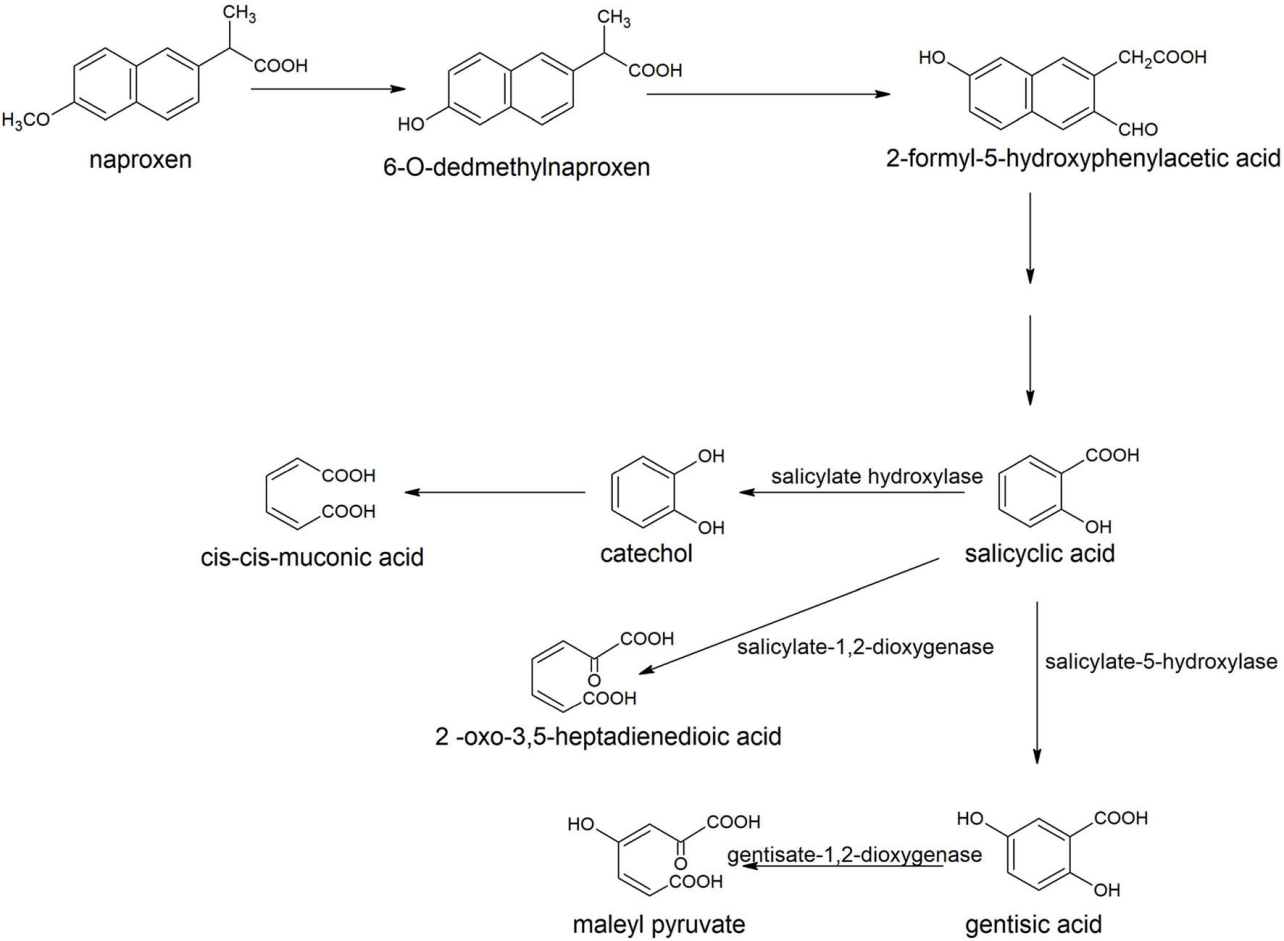
Degradation pathway of naproxen in *Bacillus thuringiensis* B1 (Górny et al., 2019).

The degradation of naproxen was initiated with the transformation of naproxen into o-desmethylnaproxen by the action of tetrahydrofolate dependent *O*-demethylase (Górny et al., 2019). The next step involved the formation of 2-formyl-5-hydroxyphenylacetic that was converted to salicylic acid (Figure 9). Salicyclic acid hydroxylated to catechol or gentisic acid or can be cleaved to 2-oxo-3, 5-heptadienedioic acid (Górny et al., 2019).

### *Bacilli*-Mediated Transformation of Heavy Metals

The bacterial remediation of heavy metals involves removals of heavy metals from aqueous solution and soil through biosorption, bioaccumulation, or biotransformation (Dixit et al., 2015). Biosorption is one of the important mechanisms for the removal of heavy metals, which involves the interaction of heavy metals with the functional groups present on bacterial surfaces (Igiri et al., 2018). Bioaccumulation is a metabolism-driven process in which the heavy metal ions pass across the cell membrane into the cytoplasm, accumulating inside the cells (Diep et al., 2018). Biotransformation involves conversation of one form of heavy metal to another form (Juwarkar and Yadav, 2010). In this subsection, the role of *Bacilli* in the bioremediation of various heavy metals is summarized.

Many *Bacilli* have been characterized for the bioreduction of chromium from Cr(VI) to Cr(III). Examples are *Bacillus* sp. strain FM1 (Masood and Malik, 2011), *Bacillus* sp. strain KSUCr9a (Ibrahim et al., 2012), *B*. *sphaericus* AND 303 (Pal et al., 2005), *Bacillus* sp. FY1 (Xiao et al., 2017), *Bacillus* sp. MNU16 (Upadhyay et al., 2017)*, B. amyloliquefaciens* (Das et al., 2014), and *B. cereus* S612 (Wang et al., 2015). Several mechanisms have been proposed for chromium reduction and removal. Chen et al. (2012c) investigated the Cr(VI) uptake mechanism in *B. cereus* that reduced Cr(VI) into Cr(III). The reduced Cr(III) was coordinated with carboxyl and amido functional groups of the bacterial cell and the Cr(III) precipitates were accumulated on bacterial surfaces. Das et al. (2014) studied the mechanism of Cr(VI) reduction in *B. amyloliquefaciens* strain CSB 9 isolated from chromite mine soil of Sukinda, India. The reduced product Cr (III) was removed via surface immobilization and accumulated inside the bacterial cells. *Bacillus* sp. ES 29 produced copper (Cu^2+^) stimulated soluble Cr(VI)-reducing enzyme that reduced Cr(VI) to Cr(III)(Camargo et al., 2003).

The lead transformation from toxic Pb(II) to non-toxic lead compounds has been investigated in a few *Bacillus* strains. Chen et al. (2016) studied the transformation of Pb(II) into nanosized rod-shaped Ca_2.5_Pb_7.5_(OH)_2_(PO_4_)_6_ crystal in B. cereus 12-2, isolated from lead-zinc mine tailings. Initially, bacterial cells rapidly absorbed Pb(II) through the synergy of electrostatic attraction, ionic exchange, and chelating activity of functional groups present in bacterial cells. In the next step, enzyme-mediated Pb(II) transformation to rod-shaped crystalline minerals occurred inside the bacteria. Govarthanan et al. (2013) isolated and characterized an autochthonous bacterium, *Bacillus* sp. KK-1 for biomineralization of Pb in mine tailings. Strain KK-1 can convert Pb(NO_3_)_2_ into lead sulfide (PbS) and lead silicon oxide (PbSiO_3_). The ability of strain KK-1 to remove Pb was investigated in mine tailings. Strain KK-1 significantly reduced the exchangeable fraction of Pb and induced calcite in the precipitation of Pb ions.

The selenium reduction from Se(IV) to Se (III) is well-studied in *Bacillus* strains. Mishra et al. (2011) reported the reduction of Se(IV) to red-element Se (III) by two strains of B. megaterium. Garbisu et al. (1995) studied the physiological mechanisms regulating the selenite reduction in *B*. *subtilis*. They concluded that the reduction mechanism involves an inducible detoxification system, which deposited elemental selenium between the cell wall and the plasma membrane. Another mechanism was observed in a selenate reducing bacterium, *B. selenatarsenatis* SF-1, isolated from selenium-contaminated sediment (Kashiwa et al., 2001). Strain SF-1 reduced selenate to selenite and subsequently to non-toxic insoluble elemental selenium using lactate as an electron donor and selenate as an electron acceptor in an anaerobic condition. Elemental selenium was deposited both inside and outside of the cells. *B. selenitireducens* produced enzymes to reduce the oxidized forms of arsenic and selenium to their less toxic reduced forms (Wells et al., 2019). *B. cereus* CM100B and *B*. *mycoidesstrain* SeITE01 produced selenium nanoparticles (SNs) by transformation of toxic selenite (${\text{SeO}}_{3}^{2-}$) anions into red elemental selenium (Se^0^) under aerobic conditions. In this mechanism, initially, ${\text{SeO}}_{3}^{2-}$ enzymatically reduced to selenium through redox reactions by the bacterial enzymes (membrane reductase) and later, selenium nanoparticles were generated due to the result of an Ostwald ripening mechanism (Dhanjal and Cameotra, 2010; Lampis et al., 2014).

The uranium transformation from U(VI) into nano-uramphite was studied in two *B. thuringiensis* strains isolated from uranium mine (Pan et al., 2015). The initial step involves the adsorption of U(VI) on the bacterial surface through coordinating with phosphate, -CH_2_, and amide groups. The next step involves the formation and accumulation of needle-like amorphous uranium compounds.

Paraneeiswaran et al. (2015) reported that *B. licheniformis* SPB-2 reduced [Co(III)–EDTA]^−^ to [Co(II)–EDTA]^2−^ which was further absorbed by strain SPG-2. B. firmus strain TE7, isolated from tannery effluent reduced Cr(VI) to Cr (III) and oxidized As(III) to As(V) (Bachate et al., 2013). *Bacillus* sp. strain A.rzi isolated from a metal-contaminated soil reduced molybdate to molybdenum blue (Othman et al., 2013). *B. thuringiensis* OSM29 isolated from the rhizosphere of cauliflower grown in soil irrigated consistently with industrial effluents was capable of removing several heavy metals including cadmium, chromium, copper, lead and nickel via biosorption (Oves et al., 2013). The biosorption capacity of the strain OSM29 for the metallic ions was highest for Ni (94%) which was followed by Cu (91.8%).

### *Bacilli*-Mediated Transformation of Azo Dyes

Azo dyes are a large group of synthetic aromatic compounds which contain one or more azo groups (-N=N-) between organic residues. Based on the number of azo linkages, azo dyes are classified as monoazo, disazo, trisazo, and polyazo (Benkhaya et al., 2020). Few examples of azo dyes are Metanil Yellow, Navy Blue 2GL, Dye Orange T4LL, Reactive Red 2, Direct Red-22, Turquoise Blue dye, and Acid Black 24. These are widely used in the textile industry that is a major source of dye contamination. During the dyeing process, the textile industry discharged ~10% of the dyes into the wastewater (Easton, 1995). Apart from the textile industry, azo dyes are also used in food, paper printing, color photography, leather, and cosmetic industries (Chang and Lin, 2001). They are widely distributed in the environment due to improper discharge of dye into wastewater. These dyes are highly toxic to plants by inhibiting their photosynthesis. In the environment, they may generate mutagenic and carcinogenic amines due to microbial transformation (Chung and Cerniglia, 1992; Weisburger, 2002; Asad et al., 2007). Dye removal is an essential step for the treatment of dye-containing wastewater (Banat et al., 1996). Microbial dye degradation process has two steps; First is dye decolorization in which azoreductase-mediated cleavage of the azo bond (—N=N—) to give aromatic amines. The second step involves the degradation of aromatic amines into non-toxic compounds. In this sub-section, the role of *Bacilli* in dye decolorization is summarized.

Many *Bacillus* strains have been characterized for decolorization of wastewater containing various azo dyes. Anjaneya et al. (2011) studied the decolorization of metanil yellow using a sulfonated azo dye decolourizing bacterium, *Bacillus* sp. AK1 that was isolated from dye contaminated soil sample collected from Atul Dyeing Industry, Bellary, India. *Bacillus* sp. AK1 decolorized metanil yellow (200 mg L^−1^) completely within 27h and transformed it into metanillic acid and *p*-aminodiphenylamine by the action of the azoreductase enzyme. Dawkar et al. (2009) studied the effects of inducers on the decolorization of a textile azo dye, navy blue 2GL by a *Bacillus* sp. VUS isolated from textile effluent contaminated soil. Strain VUS decolorized azo dye navy blue 2GL within 48 h under the static anoxic condition in yeast extract medium, whereas in the presence of CaCl_2_ it decolorized it only within 18 h. They reported that CaCl_2_ induced the activities of the enzymes involved in the decolorization of navy blue 2GL. 4-Amino-3-(2-bromo-4, 6-dinitro-phenylazo)-phenol and acetic acid 2-(-acetoxy-ethylamino)-ethyl ester were detected as the transformation products of dye decolorization. *Bacillus* sp. VUS also decolorized dye orange T4LL in static anoxic condition within 24 h and transformed it into 4-methyl-2-o-tolylazo-benzene-1,3-diamine and [3-(phenyl-hydrazono)-cyclohexa-1,4-dienyl]-methanol. Another bacterium, *B*. *licheniformis* decoulorized Reactive Red 2 and transformed it into 2, 4-dichloro-6-[(1H-indazol-5-ylimino)-methyl]-phenol, benzene sulfonamide, 1H indole and urea as final metabolites (Sudha and Balagurunathan, 2013). *B*. *firmus* immobilized within tubular polymeric gel completely decolorized 50 mg/L of CI Direct Red 80 under anoxic conditions within 12 h by transforming it into aromatic amine (Ogugbue et al., 2012). These aromatic amines were further degraded aerobically by the same strain within the subsequent 12 h.

Saleem et al. (2014) studied the effects of the various carbon sources, pH, temperature, and nitrogen sources on decolorization of pulp and paper industrial effluents by *B. cereus*. They observed that the optimum temperature and pH for decolorization were 45° C and 6.5, respectively. Maximum decolorization was observed when carbon and nitrogen sources were sucrose (0.5%) and ammonium sulfate (1%), respectively. Sharma et al. (2009) optimized process variables for decolorization of disperse yellow 211 by *B. subtilis* using Box–Behnken design and observed that the optimum conditions for maximum decolorization were 100 mg l^−1^ initial dye concentration, 7.0 pH and 32.5° C temperature. A crystal violet decolourizing bacterium, *B. subtilis* decolorized crystal violet (100 mg/L) effectively at pH 8 and temperature 35° C when starch and peptone were used as carbon and nitrogen sources, respectively (Kochher and Kumar, 2011). Gunasekar et al. (2013) reported the decolorization of reactive dye RED M5B by *B*. *subtilis* and observed that decolorization was due to the action of enzyme peroxidase produced by the organisms during its growth. Joshi et al. (2013) reported the decolorization of turquoise blue dye (Remazol Blue BB) by *B*. *megaterium* isolated from a sample collected from dye industries. This organism can decolorize turquoise blue dye up to a concentration of 5 mg/ml. Prasad and Rao (2014) reported decolorization of Acid Black 24 by *B*. *halodurans* MTCC 865 which was able to decolorize Acid Black within 6 hat pH 9 and 37° C with 5% NaCl under static conditions. Prasad and Rao (2013) reported aerobic decolorization of the textile azo dye Direct Red-22 by an obligate alkaliphilic bacterium *B. cohnii* MTCC 3616. This strain was able to decolorize Direct Red-22 (5,000 mg l^1^) with 95% efficiency at 37° C and pH 9 in 4 h under static conditions.

### *Bacilli*-Mediated Degradation of Natural Aromatic Acids

Aromatic acids are a class of chemical compounds in which an organic acid attached to the aromatic ring. Examples are phenolic acids (3-Hydroxybenzoic acid. 4-Hydroxybenzoic acid and Salicylic acid) and Hydroxycinnamic acids (cinnamic, 4-coumaric, and ferulic acids). In this subsection, the role of *Bacilli* in biodegradation of various aromatic acids is summarized. *B*. *macerans* JJ-lb degraded protocatechuate via ring cleavage and subsequent enzymatic decarboxylation of the ring fission product (Crawford et al., 1979). Initially, protocatechuate-2,3-dioxygenase catalyzes the ring cleavage of protocatechuate to 5-carboxy-2-hydroxymuconic semialdehyde that is further decarboxylated to 2-hydroxymuconic semialdehyde. Mashetty et al. (1996) reported the degradation of 3-hydroxybenzoate by a *Bacillus* sp. that utilized it as the sole source of carbon and energy. This strain metabolized 3-hydroxybenzoic acid via protocatechuic acid that was further degraded via both the *ortho-* and *meta*-cleavage pathway. The enzyme activities for 3-hydroxybenzoate 4-hydroxylase, protocatechuate 3,4-dioxygenase, and protocatechuate 4,5-dioxygenase were detected in cell-free extracts. Crawford (1976) reported degradation pathways of 4-hydroxybenzoate in *B. brevis* PHB-2, *B*. *circulans* strain 3, and *B*. *laterosporus* PHB-7a. *B. brevis* PHB-2 and *B*. *circulans* strain 3 degraded 4-hydroxybenzoate via protocatechuate that was further degraded through *ortho* cleavage pathway or *meta* cleavage pathway. *B*. *laterosporus* PHB-7a converts 4-hydroxybenzoate to gentisate, which is further degraded by the glutathione-independent gentisic acid pathway. Peng et al. (2003) reported the degradation of cinnamic, 4-coumaric, and ferulic acids by thermophilic *Bacillus* sp. B-1. Strain B-1 degraded cinnamic acid via benzoic acid that was further degraded via catechol and its ring cleavage. The 4-coumaric acid degradation proceeded via 4-hydroxybenzoic acid that was further degraded via gentisic acid and its ring cleavage. The ferculic acid metabolized via 4-hydroxy-3-methoxyphenyl-beta-hydroxypropionic acid, vanillin, and vanillic acid as the intermediates. *Bacillus* sp. DG-2 degraded 3-phenoxybenzoic acid via 3-(2-methoxyphenoxy) benzoic acid, protocatechuate, phenol, and 3,4-dihydroxy phenol.

### *Bacilli*-Mediated Degradation of Explosives

*Bacilli* play a critical role in the degradation of explosives such as nitrate esters, 2,4,6-Trinitrotoluene (TNT), Trinitrophenol (TNP). Denitration is the main step for the biodegradation of nitrate esters. Meng et al. (1995) studied the biotransformation of glycerol trinitrate by *Bacillus* sp. ATCC51912 that sequentially denitrated glycerol trinitrate to glycerol via the formation of glycerol dinitrate and glycerol mononitrate isomers. Similarly, *Bacillus* sp. ATCC51912 denitrated propylene glycol dinitrate to propylene glycol via propylene glycol mononitrate (Sun et al., 1996). Yerson and Christian (2013) isolated pentaerythritol tetranitrate (PETN)-degrading bacterium, *Bacillus* sp. J8A2 from mining environment. Strain J8A2 utilized PETN as its nitrogen source. Bacterial degradation of PENT generally initiated with sequential denitration of PENT to pentaerythritol via the intermediary formation of tri-, di-, and mononitrate pentaerythritol. An NADPH-dependent PETN reductase enzyme isolated from *Bacillus* sp. was capable of liberating nitrite from nitrate esters with the oxidation of NADPH.

*Bacillus* sp. can use TNP as a sole nitrogen source under aerobic conditions (Singh et al., 2011). TNPs has three electron-withdrawing nitro groups that prevent an initial oxidative attack on the aromatic ring. Therefore, the initial steps of TNP degradation are reductive. *Bacilli* degraded TNP by via hydrogenation to form a Meisenheimercomplex, hydride σ-complex (Singh et al., 2011).

Degradation of 2,4,6-Trinitrotoluene (TNT) by *Bacillus* sp. occurs also via the reductive route. *B*. *cereus* transformed TNT to 2,4-dinitrotoluene and 4-aminodinitrotoluene derivates and degraded 77% of 75 mg L^−1^, TNT within 96 h (Mercimek et al., 2013). Nyanhongo et al. (2008) reported that *Bacillus* sp. SF transformed TNT via an initial reduction mechanism to produce hydroxylaminodinitrotoluenes, 4-amino-2,6-dinitrotoluenes, 2-amino-4,6-dinitrotoluenes, different azoxy compounds, 2,6-diaminonitrotoluenes, and 2,4-diaminonitrotoluenes.

## Pilot Scale Studies Using *Bacilli*

For biodegradation purposes, a pilot study plays a vital role before conducting the big scale degradation studies in fields. Chopra and Kumar (2020) examined the degradation of acetaminophen (N-acetyl-para-aminophenol) by *B. drentensis* strain S1 within the the pilot-scale anaerobic batch reactor. The ideal conditions include temperature 40° C, pH 7, 300 mg/L acetaminophen, and agitation speed 165 rpm (Chopra and Kumar, 2020). 2-Isopropyl-5-methylcyclohexanone and phenothiazine were identified metabolites of the acetaminophen degradation. Sonwani et al. (2019) studied the degradation of naphthalene in a pilot-scale integrated aerobic treatment plant and catechol and 2-naphthol were detected as the major intermediate metabolites. Fujita et al. (2002) studied the removal of toxic soluble selenium (selenite/selenate) using *Bacillus* sp. SF-1 in a continuous flow bioreactor under an anoxic condition. The outcomes indicated that both selenite and selenate were reduced to elemental selenium at long cell retention times. Sundar et al. (2011) successfully demonstrated the removal of trivalent chromium using *Bacillus* biofilms through a continuous flow reactor. Pan et al. (2014) used a mixture of planktonic cells and biofilms of *B. subtilis* for successful removal of Cr(IV) from Cr(IV)-containing wastewater in 10-L pilot-scale experiment. Kim et al. (2014) treated 80 tons of groundwater containing heavy metals using immobilized dead cells of *B. drentensis* in pilot-scale study and results demonstrated over 93% removal of Cu, Cd, Zn, and Fe. Narayanan et al. (2015) reported the production of laccase from *B. subtilis* MTCC 2414 for the study of decolorization of Yellow GR, Orange 3R, and T-Blue. They used guaiacol as a substrate under Submerged Fermentation Conditions for the production of laccase, which was immobilized with sodium alginate. The immobilized laccase exhibited optimum activity at pH 7 and temperature 35° C. Results of their studies showed that immobilized laccases degraded Yellow GR (81.72%), Orange 3R (77.2%), and T-Blue (78.55%) at higher efficiency as compared to free laccase. Several researchers investigated the pilot scale-production of commercial compounds using various wastes as substrates (Mohapatra et al., 2017). Yezza et al. (2004) studied the production of *Bacillus thuringiensis*-based biopesticides in fermenters using wastewater sludge as raw materials and results demonstrated high production of pesticides. Mohapatra et al. (2017) studied bioconversion of fish solid waste into polyhydroxybutyrate using the *Bacillus subtilis*-based submerged fermentation process. Barros et al. (2008) reported the production of biosurfactant by *Bacillus subtilis* on a pilot scale using cassava wastewater as substrate.

## Advanced Technologies for Bioremediation of Xenobiotic Compounds and Heavy Metals Using *Bacilli*

This section briefly describes various current technologies used to enhance the bioremediation of xenobiotic compounds and heavy metals.

### Metagenomics

Several xenobiotic-degrading enzymes stay undiscovered in light of the fact that a greater part of bacteria (99%) remain uncluturable in laboratory (Arora et al., 2010). In such a case, metagenomics plays a vital role to investigate novel microbial enzymes from whole network of microbial community. The metagenomic approach includes (i) the isolation and purification of DNA from a sample, (ii) cloning of DNA into appropriate vectors, (iii) the transformation of host cells with construct and (iv) functional and sequence based screening of constructed clones (Arora et al., 2010). The sequence-based approaches depend on already known sequences of the target gene and utilize bioinformatics tools. However, the function-based approaches do not include the involvement of metagenomic derived sequences and, in this way, may prompt to the invention of novel genes with desired functions. Several enzymes involved in biodegradation of various xenobiotic compounds have been identified by metagenomic studies of several environmental samples. Sidhu C. et al. (2019) identified novel 2,3-dihydroxybiphenyl 1,2-dioxygenase (BphC-SD3) and catechol 2,3-dioxygenase (C23O-RW1) from the metagenomic DNA isolated from sludge and river water samples. These enzymes were clones, expressed and purified to monitor their abilities to degrade various aromatic compounds. BphC-SD3 specifically oxidized 2,3-dihydroxybiphenyl, catechol, and 3-methylcatechol, whereas C23O-RW1 oxidized catechol, 4-chlorocatechol, 2,3-dihydroxybiphenyl and 3-methylcatechol. Suenaga et al. (2007) studied extradiol dioxygenases diversity in activated sludge used to treat coke plant wastewater by a metagenomic approach and identified 38 new extradiol dioxygenases that formed a new subfamily of extradiol dioxygenases. Singh et al. (2010) identified two flavin monooxygenases from an effluent treatment plant sludge metagenomic library which were involved in the oxidation of indole to a mixture of indigo and indirubin pigments. Nagayama et al. (2015) identified a multicomponent hydroxylase involved in the phenol degradation from a metagenomic library derived from soil sample artificially contaminated with aromatic compounds. Choi et al. (2018) identified and characterized the first metagenome-derived toxoflavin-degrading enzyme that was involved in biodegradation of toxoflavin and its derivatives including methyltoxoflavin, fervenulin, and reumycin. Ye et al. (2010) identified a muti-copper oxidase with laccase activity from activity-based functional screening of a metagenomic library from mangrove soil. The characteristic feature of this laccase was its strong alkaline activity and its high solubility.

### Rational Designing

This protein engineering approach requires the knowledge of protein structure, function and mechanism to improve enzyme properties. Several xenobiotic-degrading enzymes of *Bacilli* have been improved using rational designing approach. Best studied example is laccase enzyme that catalyzes the oxidation of a variety of xenobiotic compounds, including diphenols, polyphenols, diamines, aromatic amines, and synthetic dyes. Mollania et al. (2011) used rational design approach to increase the thermal stability of laccase enzyme of *Bacillus* sp. HR03. They substituted Glu188 residue with 2 positive (Lys and Arg) and one hydrophobic (Ala) residues to obtain mutants. All variants exhibited strong thermal stability and thermal activation as compared to the wild-type. The 3-fold higher thermal activation and higher *T*_50_ (5° C) as compared to native enzyme was observed in the case of the Glu188Lys variant (Mollania et al., 2011). Rasekh et al. (2014) increased the tolerance of this laccase toward organic solvents by substitution of the Glu188 residue with non-polar (Ala, Ile, Leu, and Val) and positively charged (Lys and Arg) residues. All variants showed higher C_50_ values (organic solvent concentration at which 50% of enzyme activity remains) as compared to the wild type. Non-polar amino acid substitutions created more efficient mutants as they exhibited significantly increased C_50_ value and decreased thermo inactivation rate in the presence of organic solvents (Rasekh et al., 2014).

Another example of rational design to improve the enzyme activity is cytochrome P450 monooxygenase from *Bacillus megaterium* 3 (P450 BM3). Carmichael and Wong (2001) reported double mutation in P450 BM3 at R47L and Y51F to enhance its oxidation activity toward phenanthrene and fluoranthene. The mutants showed 40-folds and 10-folds oxidation activity toward phenanthrene and fluoranthene. Li et al. (2001) reported oxidation of polycyclic hydrocarbons such as naphthalene, fluorene, acenaphthene, acenaphthylene, and 9-methylanthracene by triple mutant of P450 BM3 at A74G/F87V/L188Q sites.

### Directed Evolution

Directed evolution is an approach of protein engineering to improve the efficiency of proteins without a prior knowledge of amino acid sequences. It is based on the Darwinian principle of evolution and involves (i) the use of rapid molecular manipulations to mutate the target gene and (ii) the subsequent selection of the improved variants by screening (Arora et al., 2010). Using directed evolution, many xenobiotic-degrading genes have been improved for their properties. Best studied example is cytochrome P450 monooxygenase from *Bacillus megaterium* 3 (P450 BM3) that involves in oxidation of various aromatic compounds. Sideri et al. (2013) used directed evolution to generate mutants of P450 BM3 to hydoxylate chrysene and pyrene. Two rounds of random mutagenesis by error–prone PCR were used to generate mutants. Three mutants exhibited hydroxylation of chrysene and pyrene. These mutants hydroxylated chrysene in different positions and hydroxylate pyrene to 1-hydroxypyrene. Santos et al. (2019) reported that directed evolution of P450 BM3 to improve the hydroxylation activity toward six o-heterocycles; benzo-1,4-dioxane, phthalan, isochroman, 2,3-dihydrobenzofuran, benzofuran, and dibenzofuran. They screened in-house libraries of P450 BM3 to generate P450 BM3 CM1 (R255P/P329H) that was further underwent error–prone PCR, generating P450 BM3 GS2 (R255S/P329H/F331L). Another error-prone PCR of P450 BM3 GS-2 generated P450 BM3 GS3 (I122V/R255S/P329H/F331L). In next step, P450 BM3 WT was subjected to single site saturation mutagenesis (SSM) in the four identified positions and double SSM at positions I122 and R255, which provided the most active variants, P450 BM3 R255G and R255L.

### Recombinant DNA Technology or Genetic Engineering

Genetic engineering or recombinant DNA technology includes multiple techniques used to cut up and join together DNA from various biological sources, and to introduce the resulting hybrid DNA into an organism so as to create new combinations of heritable genetic material (Rosenberg, 2017). Genetic engineering is a promising technique to enhance the potentials of microorganisms for the bioremediation of environmental pollutants (Ezezika and Singer, 2010). Genetically engineered bacteria are considered as potential candidates for bioremediation applications in soil, groundwater, and activated sludge (Sayler and Ripp, 2000). A list of few genetically engineered bacteria with their bioremediation applications is presented in Table 2.

**Table 2:** A list of few genetically engineered bacteria invloved in bioremediation.

**Genetically engineered bacteria**	**Compound/heavy metal**	**Properties/application**	**References**
*Bacillus subtilis* 168	Arsenic	Expressed the arsenite S-adenosylmethionine methyltransferase gene from thermophilic algae, *Cyanidioschyzon merolae*. This bacterium involved in arsenic methylation and volatilization	Huang et al., 2015
*Rhodopseudomonas palustris*	Mercury	Expressed mercury transport system and metallothionein for Hg2+ uptake	Deng and Jia, 2011
*Escherichia coli*	Nickel	Expressed nickel-affinity transmembrane protiens and metallothionein for Ni2+ bioaccumulation	Deng et al., 2013
*Pseudomonas putida* MC4-5222	1,2,3-Trichloropropane (TCP)	Expressed the haloalkane dehalogenase (DhaA31). More than 95% degradation of TCP was observed	Samin et al., 2014
*Pseudomonas fluorescens*	Hexahydro-1,3,5-trinitro-1,3,5-triazine (RDX)	Expressed the RDX-metabolizing enzyme XplA to degrade RDX in the rhizosphere	Lorenz et al., 2013
*Pseudomonas putida* KTUe	Organophosphates, pyrethroids, and carbamates	A scarless genome editing strategy was used to insert four pesticide degrading genes, *vgb*, and *gfp*. This bacterium completely degraded methyl parathion, chlorpyrifos, fenpropathrin, cypermethrin, carbofuran and carbaryl when concentration was 50 mg/L	Gong et al., 2018
*Cupriavidus necator* JMP134-ONP	Nitrophenols	Inserted ortho-nitrophenol degradation operon (*onpABC* gene cluster). This bacterium was able to degrade two isomers of nitrophenols	Hu et al., 2014

Even though several genetically engineered *Bacilli* have been constructed for various industrial applications (Wang et al., 2006; Drejer et al., 2020), the bioremediation applications of genetically engineered *Bacilli* is very limited. Huang et al. (2015) constructed a genetically engineered *B. subtilis* 168 expressing the arsenite S-adenosylmethionine methyltransferase gene of thermophilic algae for bioremediation of arsenic. This genetically engineered bacterium was able to convert the inorganic As into dimethylarsenate and trimethylarsine oxide via methylation, and also able to volatilize substantial amounts of dimethylarsine and trimethylarsine (Huang et al., 2015). The rate of As methylation and volatilization increased with temperature from 37 to 50° C. However, wild type *B. subtilis* 168 lacks the properties of methylation and volatilization.

### Genome-Editing Technologies

Genome-editing technologies are currently using to manipulate DNA by the engineered nucleases or molecular scissors, which have a wide range of applications in research fields of plants, animals, and microorganisms (Jaiswal et al., 2019). The process of genome editing is generally performed by genome editing tools and involves following steps (i) double standard break in targeted gene sequence (ii) repaired by homologous recombination using self-designed guide sequence complementary to targeted gene sequence (iii) error-prone non-homologous end joining (Jaiswal et al., 2019). The aim of using gene-editing tools is to develop a microbe with great potentials. Jaiswal et al. (2019) describe the role of the gene-editing tools such as Transcription-activators like effector nucleases (TALEN), clustered regularly interspaced short palindromic repeats (CRISPR-Cas), and zinc finger nucleases (ZFNs) to design bacteria with improved metabolic capabilities for enhancing the bioremediation of environmental pollutants.

### Genomics

Genomic studies are a powerful tool for the study of microorganisms capable of degrading environmental pollutants (Rodríguez et al., 2020). Next-Generation sequencing technology has been widely used for the whole-genome sequences of various organisms. The whole genomes of several xenobiotic-degrading *Bacilli* have been sequenced using Next-Generation sequencing technology, and several genes and proteins involved in biodegradation have been identified through gene predictions and annotation of the *Bacilli* genomes. Hossain et al. (2020) identified chromate transporters in the genome of a chromium-reducing bacterium, *B. cereus* TN10 isolated from tannery effluent. Chromate transporters are involved in chromium resistance and play a role in the efflux of cytoplasmic chromate. He et al. (2010) identified a putative chromate transport operon, two chromate transporters, azoreductase gene, and four nitroreductase genes in *Bacillus cereus* SJ1 which may be involved chromate resistance and chromate reduction. The genome of *B. cereus* S612 contains genes encoding multidrug efflux pumps and reductases that are potentially related to chromium resistance and reduction (Wang et al., 2015). Genome analysis of zearalenone-degrading *Bacillus velezensis* ANSB01E revealed the presence of genes coding peroxiredoxin and alpha/beta hydrolase, which may be involved in zearalenone degradation (Guo et al., 2020).

### Bioinformatics Tools

Bioinformatics approaches including biodegradative databases, pathway prediction systems, and protein-structure predicting tools may be used for biodegradation studies (Arora and Bae, 2014). Biodegradative databases provide information about pollutants, their degradation pathways, bacteria, genes, and enzymes in their degradation (Arora and Bae, 2014). Examples of these databases are the EAWAG Biocatalysis/Biodegradation Database (EAWAG-BBD), a database of biodegradative oxygenases (OxDBase), Biodegradation Network-Molecular Biology database (Bionemo), MetaCyc, and BioCyc (Arora and Bae, 2014). The structure of enzymes involved in biodegradation of environmental pollutants in *Bacilli* can be predicted by online structure prediction tools such as Iterative Threading Assembly Refinement server (I-TASSER) (Yang and Zhang, 2015), SWISS-MODEL (Waterhouse et al., 2018), and optimized protein fold RecognitION (ORION) (Ghouzam et al., 2015).

## Conclusion

Many *Bacilli* have been isolated and characterized for degradation of various environmental pollutants including chloronitrophenols, dyes, drugs, pesticide, explosives, polycyclic aromatic compounds, heterocyclic aromatic compounds, and heavy metals. The biochemical characterization of degradation pathways of various environmental pollutants was extensively studied in *Bacilli*. The genes involved in the degradation of various xenobiotic compounds have been identified from the genome sequences of various xenobiotic degrading *Bacilli*. Further studies on cloning and expression of these genes would be useful to understand the mechanism of biodegradation. The construction of genetically engineered *Bacilli* with improved degradation efficiency will be useful for biodegradation applications. Furthermore, genome editing tools may be used to develop more efficient *Bacilli* for the bioremediation of pollutants. Bioinformatics tools such as databases, pathway prediction systems, and protein structure predicting tools are useful to determine the fate of environmental pollutants in the fields.

## Author Contributions

The author confirms being the sole contributor of this work and has approved it for publication.

## Conflict of Interest

The author declares that the research was conducted in the absence of any commercial or financial relationships that could be construed as a potential conflict of interest.
